# Author Correction: Avocado: a multi-scale deep tensor factorization method learns a latent representation of the human epigenome

**DOI:** 10.1186/s13059-021-02470-4

**Published:** 2021-09-03

**Authors:** Jacob Schreiber, Timothy Durham, Jeffrey Bilmes, William Stafford Noble

**Affiliations:** 1 Paul G. Allen School of Computer Science and Engineering, University of Washington, Seattle, USA; 2 Department of Genome Sciences, University of Washington, Seattle, USA; 3 Department of Electrical Engineering, University of Washington, Seattle, USA

**Correction to: Genome Biol 21, 81 (2020)**

**https://doi.org/10.1186/s13059-020-01977-6**

Following publication of the original article [1], the following error was identified in the Results section, under the heading *Avocado imputes epigenomic tracks more accurately than prior methods*.

“Conversely, ChromImpute performs the best on MSE1obs (Avocado/ChromImpute p value = 2.37e −22, PREDICTD/ChromImpute p value = 2.85e −12) but the worst on MSE1imp, suggesting that it may over-call peaks. Additionally, Ernst and Kellis proposed six other evaluation performance measures, which show similar trends as the MSE1imp metric (Additional file 2: Fig. S2).”

The second sentence above should read (with the changed text in **bold**)

“Additionally, Ernst and Kellis proposed six other evaluation performance measures, which show similar trends as the **MSE1obs** metric (Additional file 2: Fig. S2).”

Because this was a typo, the primary results and conclusions in the paper still hold. However, to improve the clarity of our claims in light of this correction, we would like to make two additional edits:

In the Background section, we would like to change:

“Using data from the Roadmap Epigenomics Consortium, we demonstrate that Avocado yields imputed values that are more accurate than those produced by either ChromImpute [6] or PREDICTD [5], as measured by multiple performance measures.”

to

“Using data from the Roadmap Epigenomics Consortium, we demonstrate that Avocado yields imputed values that are more accurate than those produced by either ChromImpute [6] or PREDICTD [5], as measured by multiple performance measures **based on MSE**.”

In the Conclusion section, we would like to change:

“This latent representation is trained to impute genome-wide epigenomics experiments, and we find that the resulting model outperforms prior methods at that task.”

to

“This latent representation is trained to impute genome-wide epigenomics experiments, and we find that the resulting model outperforms prior methods at that task according to several performance measures **based on MSE**.”

The original article [1] has been corrected.

## References

[1] Schreiber J, Durham T, Bilmes J, Noble WS (2020). Avocado: a multi-scale deep tensor factorization method learns a latent representation of the human epigenome. Genome Biol.

