# Supplementary material for: Avocado: a multi-scale deep tensor factorization method learns a latent representation of the human epigenome
Source: Genome Biol. 2020 Mar 30;21:81. doi: 10.1186/s13059-020-01977-6 (PMC7104480; doi:10.1186/s13059-020-01977-6)
Supplement: Supplementary file 1 — Additional file 1 Hyperparameter selection. Details related to empirically choosing the hyperparameters for the Avocado model using a randomized grid-search procedure. [file 13059_2020_1977_MOESM1_ESM.pdf]

Additional File 1: Hyperparameter selection

Jacob Schreiber[1], Timothy Durham[2], Jeffrey Bilmes[1,3], and William Stafford Noble[1,2]

[1]Paul G. Allen School of Computer Science and Engineering, University of Washington, Seattle, USA
[2]Department of Genome Sciences, University of Washington, Seattle, USA
[3]Department of Electrical Engineering, University of Washington, Seattle, USA

November 30, 2019

Avocado's model has seven structural hyperparameters: the number of latent factors representing cell types, assay types, and the three scales of genomic positions, as well as two parameters (number of layers and number of nodes per layer) for the deep neural network.

We optimized these hyperparameters via random search. The search considered the following grid of values: cell type factors $\in$ (16, 32, 64, 128, 256), assay factors $\in$ (16, 32, 64, 128, 256), 25 bp resolution genome factors $\in$ (5, 10, 15, 20, 25), 250 bp resolution genome factors $\in$ (10, 20, 30, 40, 50), 5 kbp resolution genome factors $\in$ (15, 30, 45, 60, 75), number of layers in the neural network $\in$ (0, 1, 2, 3, 4), and number of neurons in the neural network $\in$ (128, 256, 512, 1024, 2048). Note that setting the number of layers to 0 corresponds to training a linear regression model on top of the learned factors. These ranges were selected based on experimental results from Durham et al. [1], suggesting that 100 latent factors for each of the three axes performed well. In this grid we trained 1,000 models out of a possible ∼61,000. Each model was trained on the ENCODE Pilot Regions, which are comprised of 44 regions of 0.5-2 Mb length that jointly make up approximately 1% of the full genome. The data were split into a training set of 764 tracks, a validation set of 100 tracks, and a test set of 150 tracks. We selected the final set of hyperparameters based on performance on the validation set, as measured by mean-squared error (MSE).

The different hyperparameter settings displayed a wide variance in performance, with most performing better than ChromImpute and many performing better than PREDICTD on the validation set (Additional file 1: Figure S1). Once the hyperparameters were set, the model was then retrained on both the training and validation sets and tested on the held-out test set. Note that the training, validation, and test sets used here correspond to the same splits used for the PREDICTD approach. The resulting model had a MSE of 0.1130 on the test set, which represents an 18.5% improvement over ChromImpute (MSE 0.1387) and a 4.9% improvement over PREDICTD (MSE 0.1188).

We next investigated the effect that each hyperparameter had on the overall predictive performance of Avocado. To do this, we considered each hyperparameter individually and, for each value that the hyperparameter could take, we plotted the MSE of each model that used that value (Additional file 1: Figure S2). The clearest trend was that the performance of the model increased as the size of the neural network increased, both in terms of the number of layers and the number of neurons per layer. In contrast, the number of latent factors did not show a clear trend of improvement over any of the three axes.

To attempt to better understand where the allocation of parameters was most beneficial, we considered performance when compared to the total number of parameters in the neural network and when compared to the total number of parameters in the embedding matrices (Additional file 1: Figure S3). We see that the validation set error decreases steadily with an increase in the number of network parameters until leveling off around 10^7 parameters. In particular, having no hidden layer, i.e., learning a linear regression on top of the tensor factorization, leads to very poor models. However, adding more than two layers does not yield much gain. When considering the number of parameters at each genomic position in the tensor factorization, we see no similar trend of increased complexity leading to increased performance. We focus on the number of parameters per genomic position rather than the total number of parameters in the model

because otherwise the genomic axis would dominate. We can see that having one hidden layer improves model performance; however, we do not see a trend where deeper models are able to better utilize more complex tensor factorization models. Overall, these results suggests that the use of a neural network coupled with the tensor factorization can significantly boost the performance of the model, but that the model is not very sensitive to the complexity of the tensor factorization component.

References

[1] T. J. Durham, M. W. Libbrecht, J. J. Howbert, J. A. Bilmes, and W. S. Noble. PREDICTD: PaRallel Epigenomics Data Imputation with Cloud-based Tensor Decomposition. *Nature Communications*, 9, 2018.

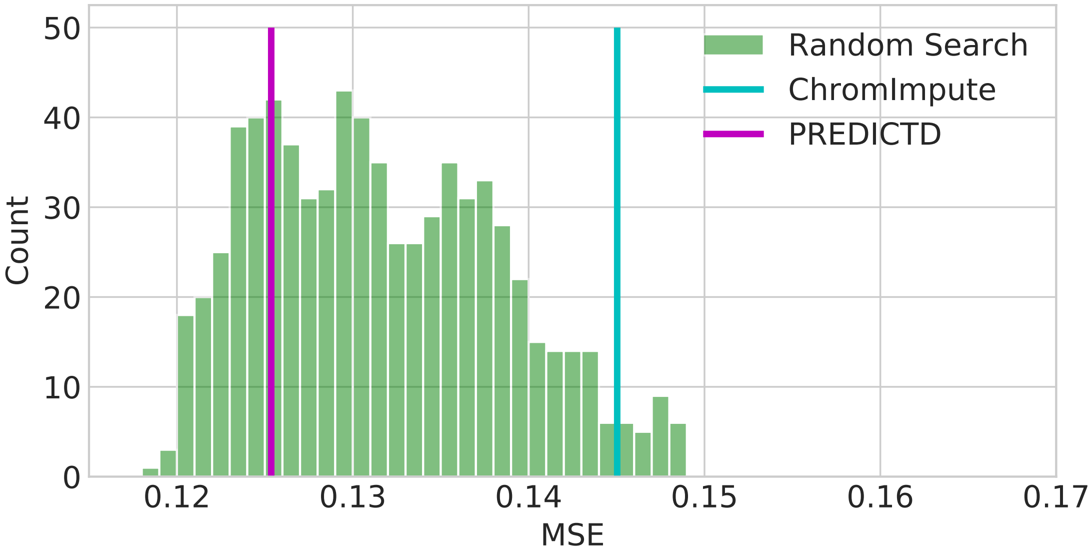

Figure S1: **Random search results on ENCODE pilot regions.** The figure plots a histogram of Avocado validation set MSE values across each hyperparameter setting. For reference, MSE values on the same data set for ChromImpute and PREDICTD are depicted as vertical lines.

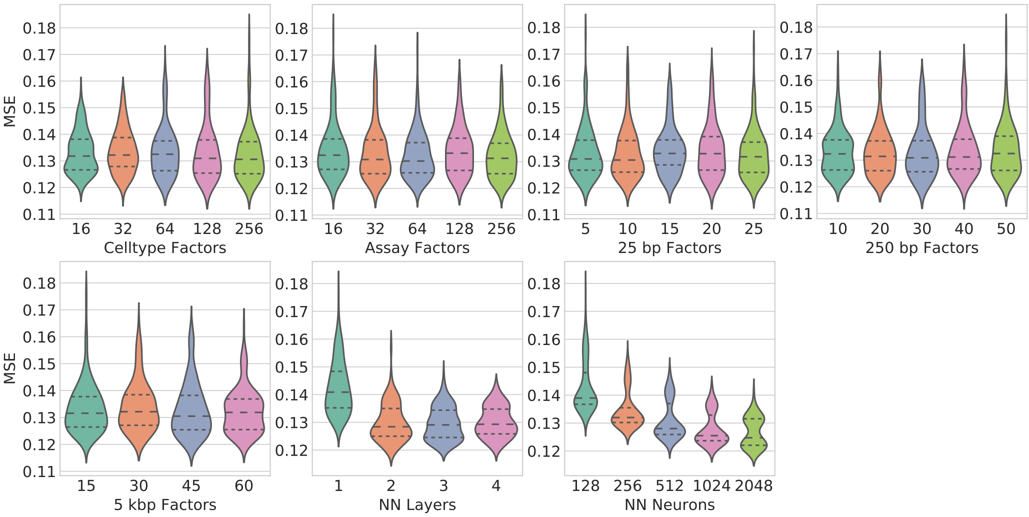

Figure S2: **The performance of the Avocado models learned during random search when stratified by values for each hyperparameter individually.** Each panel shows results for all models that had at least one hidden layer in the neural network. The median is indicated in each violin plot with the longer dashed lines, with the shorter dashed lines indicating the inter-quartile range. The performance seems to be fairly constant across hyperparameter values, except for those hyperparameters related to the neural network. Increasing the number of neurons per layer seemed to increase performance consistently, whereas past two layers the model did not appear to learn significantly more. Models with no hidden layers are not shown, because their performance was uniformly poor.

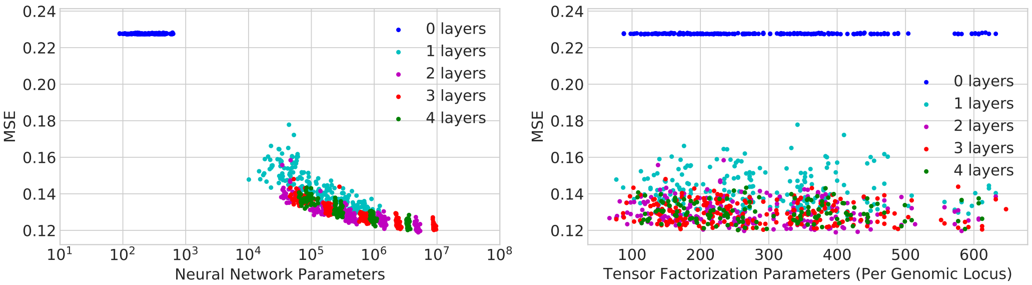

Figure S3: **The number of parameters in each model considered as a part of the random search procedure compared to validation set performance for both the neural network and the tensor factorization aspects.** Left: The trend appears to be that the greater the number of parameters, the better the performance of the model. Models with no hidden layers still have parameters in the form of a linear regression on top of the tensor factorization. The models are colored by the number of layers that they have. Right: The number of parameters in the tensor factorization component at each genomic position. This corresponds to the number of cell type factors plus the number of assay factors plus the number of genomic factors at each resolution. The models are colored by the number of layer in the neural network.
