# Supplementary material for: Avocado: a multi-scale deep tensor factorization method learns a latent representation of the human epigenome
Source: Genome Biol. 2020 Mar 30;21:81. doi: 10.1186/s13059-020-01977-6 (PMC7104480; doi:10.1186/s13059-020-01977-6)
Supplement: Supplementary file 2 — Additional file 2 Supplementary Figures and Table. Supplementary figures, tables, and follow-up analyses. [file 13059_2020_1977_MOESM2_ESM.pdf]

Additional File 2: Statistical significance analysis

Jacob Schreiber[1], Timothy Durham[2], Jeffrey Bilmes[1,3], and William Stafford Noble[1,2]

[1]Paul G. Allen School of Computer Science and Engineering, University of Washington, Seattle, USA
[2]Department of Genome Sciences, University of Washington, Seattle, USA
[3]Department of Electrical Engineering, University of Washington, Seattle, USA

November 30, 2019

	**Avocado / ChromImpute**	**Avocado / PREDICTD**	**PREDICTD / ChromImpute**
**MSEglobal**	1.21e-59	**4.51e-01**	2.00e-60
**MSE1imp**	1.97e-152	2.60e-10	1.95e-151
**MSE1obs**	2.37e-22	9.13e-06	2.85e-12
**GWcorr**	7.96e-119	2.12e-05	8.53e-110
**match1**	1.59e-138	1.71e-05	3.38e-105
**catch1obs**	1.04e-154	8.45e-50	3.79e-90
**catch1imp**	3.44e-68	1.63e-09	2.16e-51
**aucobs1**	5.00e-96	3.59e-52	4.55e-58
**aucimp1**	2.60e-25	9.22e-04	2.29e-18
**MSEProm**	3.98e-32	8.73e-05	1.04e-25
**MSEGene**	1.09e-49	**8.75e-01**	7.66e-48
**MSEEnh**	1.72e-30	1.50e-04	3.25e-23

Table S1: **Statistical significances of imputation performance measures.** Unadjusted p-values from a two-sided paired t-test that compares the average metric value across all 1,014 tracks of data for each pair of imputation methods and performance metric. The two highlighted values are the only two >0.01, indicating that all other comparisons result in statistically significant differences between the two methods.

	**B**	**R**	**CI**	**P(I)**	**A(I)**	**P(LF)**	**A(LF)**	**FRC**
**Baseline**	—							
**Roadmap**	0.0	—						
**ChromImpute**	0.0	5.31e-135	—					
PREDICTD (I)	0.0	2.48e-27	1.34e-99	—				
Avocado (I)	0.0	7.99e-116	1.25e-01	5.08e-75	—			
PREDICTD (LF)	0.0	1.19e-107	7.57e-02	2.66e-71	7.59e-01	—		
Avocado (LF)	0.0	4.62e-153	1.91e-76	8.33e-134	3.13e-104	3.86e-101	—	
FRC	0.0	2.52e-168	1.84e-69	1.59e-153	1.25e-96	2.13e-93	9.75e-21	—

Table S2: **Statistical significances of performance when predicting gene expression.** Unadjusted p-values from a two-sided paired t-test that compares the average precision across all 20 folds from all 47 cell types for a total of 940 measurements. Column names are abbreviated versions of the row names, in the same order. "(I)" stands for "imputations," "(LF)" stands for "latent factors," and "FRC" stands for "full Roadmap compendium." P-values >0.01 are in boldface.

	B	R	CI	P(I)	A(I)	A(LF)	(LF)	FRC
**Baseline**	—							
**Roadmap**	2.46e-22	—						
**ChromImpute**	1.15e-23	3.29e-10	—					
**PREDICTD (I)**	2.82e-32	1.66e-08	**0.0127**	—				
**Avocado (I)**	9.56e-19	7.4e-16	0.000176	**0.502**	—			
**PREDICTD (LF)**	9.35e-32	1.31e-20	1.34e-25	8.11e-26	9.51e-28	—		
**Avocado (LF)**	9.45e-32	9.54e-26	1.53e-26	8.33e-27	9.32e-27	6.97e-18	—	
**FRC**	1.57e-30	2.35e-09	2.02e-19	1.17e-18	2.43e-22	1.25e-12	1e-24	—

Table S3: **Statistical significances of performance when predicting promoter-enhancer interactions.** Unadjusted p-values from a two-sided paired t-test that compares the average precision across all 20 runs from all 4 cell types for a total of 80 measurements. Column names are abbreviated versions of the row names, in the same order. "(I)" stands for "imputations," "(LF)" stands for "latent factors," and "FRC" stands for "full Roadmap compendium." P-values >0.01 are in boldface.

	B	R	CI	P(I)	A(I)	P(LF)	A(LF)	FRC
**Baseline**	—							
**Roadmap**	6.91e-143	—						
**ChromImpute**	2.42e-149	3.8e-13	—					
**PREDICTD (I)**	6.93e-146	7.04e-22	2.13e-20	—				
**Avocado (I)**	1.1e-150	1.48e-21	7.83e-09	4.57e-09	—			
**PREDICTD (LF)**	5.37e-154	2.35e-22	2.73e-62	9.98e-75	3.77e-83	—		
**Avocado (LF)**	5.53e-154	2.47e-22	4.23e-58	6.26e-76	1.78e-74	0.00406	—	
**FRC**	6.64e-156	5.33e-70	3.52e-95	5.85e-82	1.96e-97	1.73e-69	2.8e-63	—

Table S4: **Statistical significances of performance when predicting replication timing.** Unadjusted p-values from a two-sided paired t-test that compares the average precision across all 20 runs from all 5 cell types for a total of 100 measurements. Column names are abbreviated versions of the row names, in the same order. "(I)" stands for "imputations," "(LF)" stands for "latent factors," and "FRC" stands for "full Roadmap compendium."

	B	R	CI	P(I)	A(I)	P(LF)	A(LF)	FRC
**Baseline**	—							
**Roadmap**	3.76e-50	—						
**ChromImpute**	2.89e-47	5.04e-21	—					
**PREDICTD (I)**	3.17e-48	2.80e-29	1.18e-08	—				
**Avocado (I)**	3.79e-48	2.17e-12	9.62e-06	2.15e-17	—			
**PREDICTD (LF)**	7.67e-53	4.69e-02	6.92e-27	1.28e-37	1.75e-18	—		
**Avocado (LF)**	6.15e-54	6.13e-08	4.39e-39	1.34e-49	1.32e-34	2.40e-04	—	
**FRC**	3.54e-56	6.72e-41	4.26e-62	7.37e-61	2.07e-55	6.94e-39	1.85e-33	—

Table S5: **Statistical significances of performance when predicting FIREs** Unadjusted p-values from a two-sided paired t-test that compares the average precision across 20 folds from all 7 cell types, for a total of 140 measurements. Column names are abbreviated versions of the row names, in the same order. "(I)" stands for "imputations," "(LF)" stands for "latent factors," and "FRC" stands for "full Roadmap compendium." P-values >0.01 are in boldface.

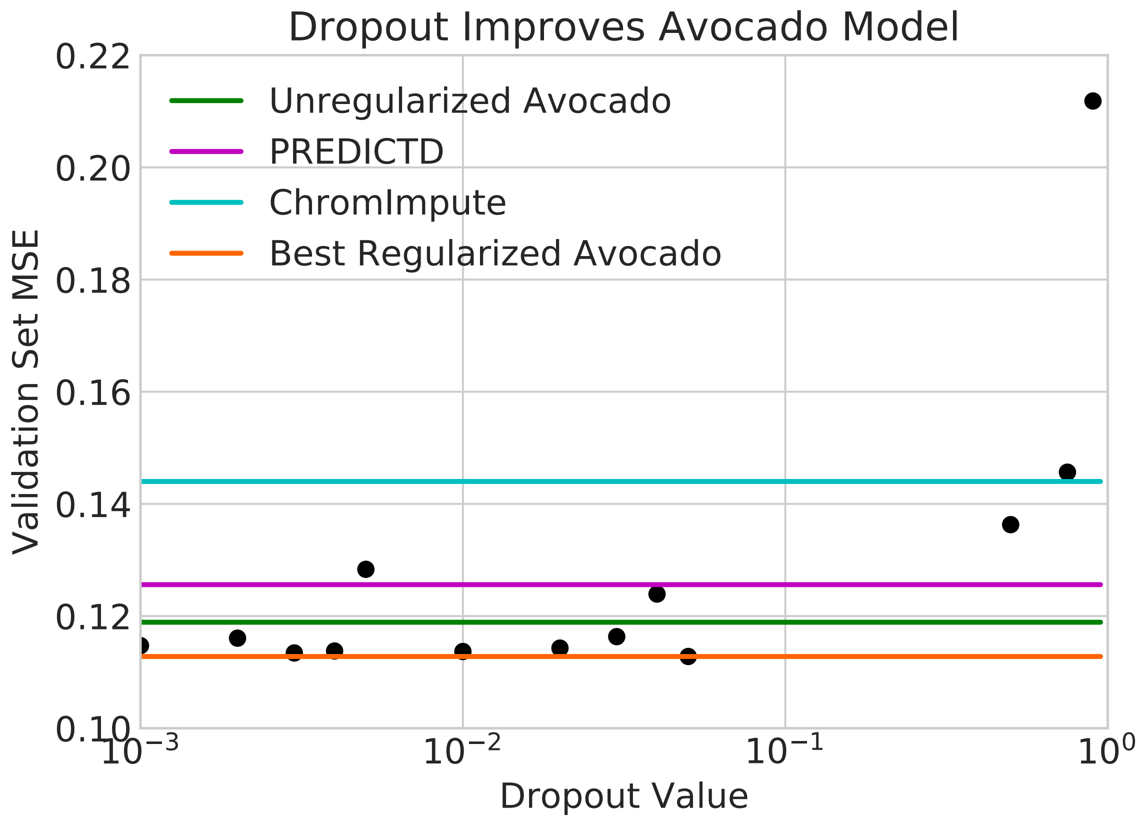

Figure S1: **Dropout improves the validation set performance of Avocado.** Each point corresponds to the performance of an Avocado model trained with a given dropout probability in the two hidden layers. The best performing model (in orange) outperforms not only the unregularized model (in green) but further improves over PREDICTD (in magenta) and ChromImpute (in cyan).

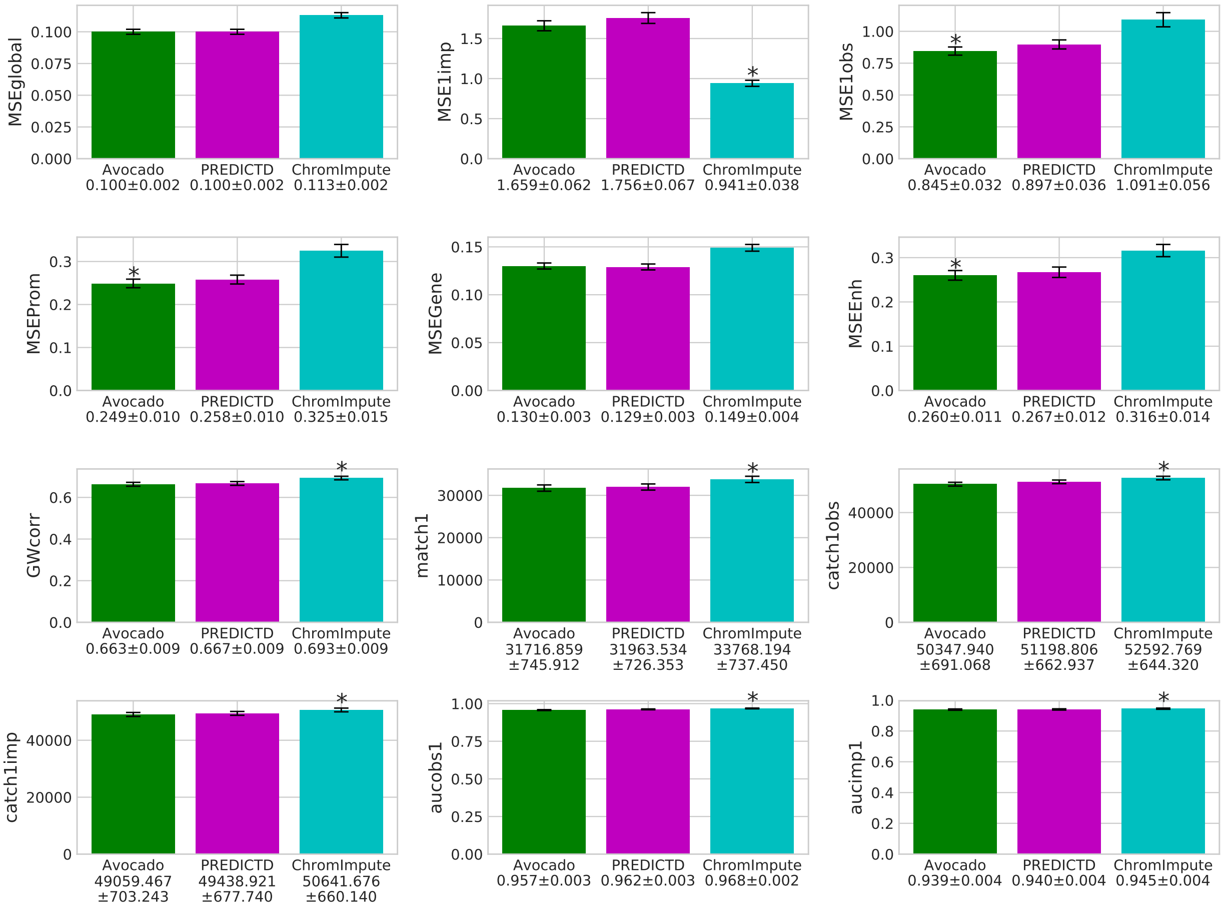

Figure S2: **Twelve performance measures evaluated across the full genome for each imputation approach**. Each panel plots the value of a specified performance measure (y-axis), averaged across all 1,014 tracks. Nine of the performance measures correspond to those proposed by either Durham et al. or Ernst and Kellis. Briefly, MSEglobal is the MSE across the full span of the genome; MSE1imp is the MSE in the top 1% of genomic positions as ranked by the observed signal value; MSE1imp is the MSE in the top 1% of as ranked by the imputed signal value for each approach separately; MSEProm is the MSE of all tracks in promoter regions; MSEGene is the MSE of all tracks in gene bodies; MSEEnh is the MSE of all tracks in enhancers; GWcorr is the Pearson correlation across the full span of the genome; match1 is the number of genomic positions in the top 1% as ranked by observed signal value that are also in the top 1% as ranked by imputed signal value; catch1obs is the number of genomic positions in the top 1% as ranked by observed signal that are in the top 5% of genomic positions as ranked by imputed signal value; catch1imp is as catch1obs but reversed; aucobs1 is the area under the receiver operator characteristics curve (AUROC) when using the imputed signal to recover the top 1% as ranked by observed signal value; and aucimp1 is as aucobs1 but reversed. Error bars display the 95% confidence interval. The best performing approach for each performance measure is denoted with an asterisk above the bar if that result is statistically significant when compared to the next highest performing approach, i.e., p-value < 0.01 on a two sided paired t-test, adjusted for the three comparisons.

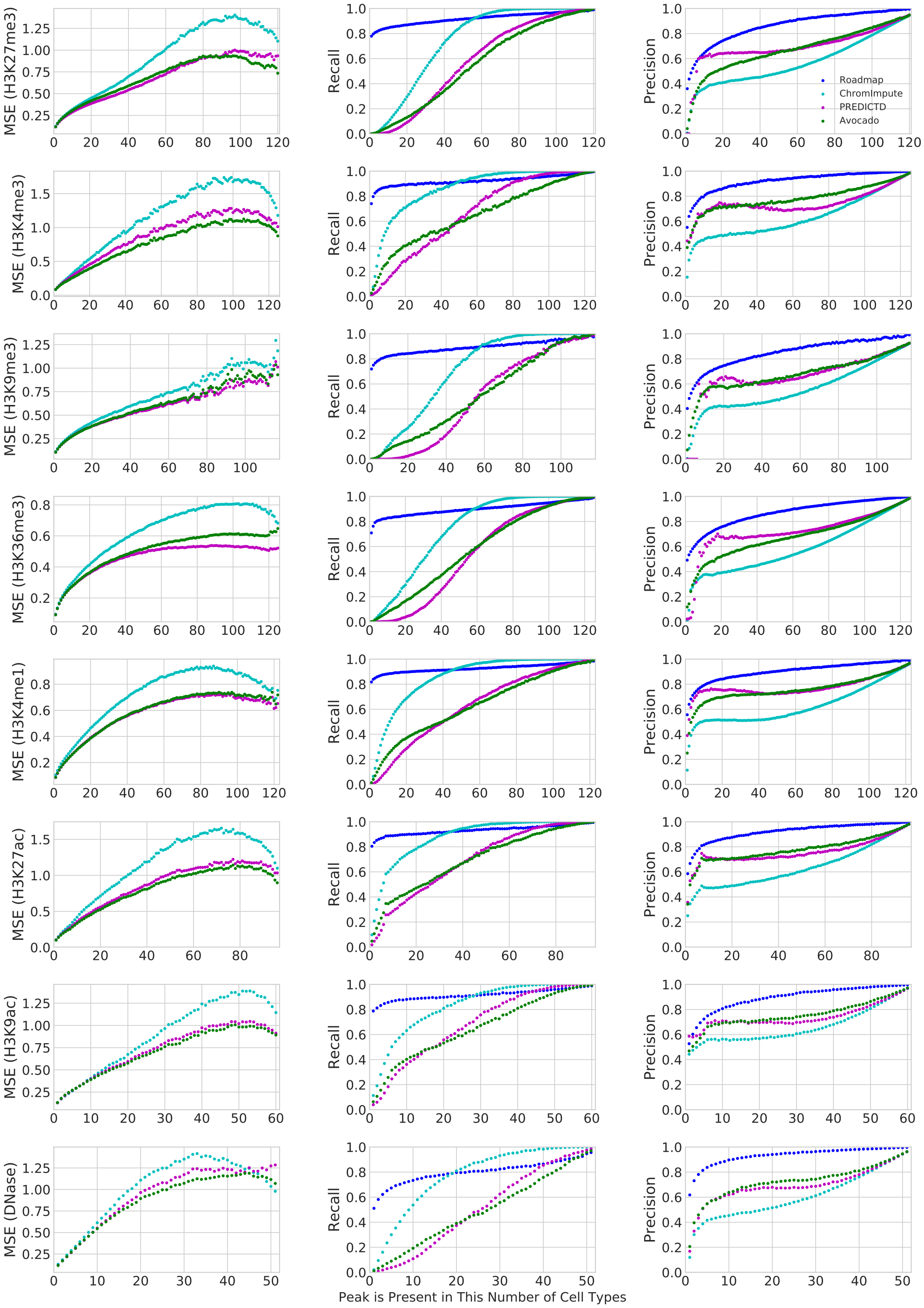

Figure S3: **Ability to recover cell type-specific peaks.** Each panel plots, for a given assay type, the MSE (left column), recall (middle column) or precision (right column) as a function of the number of cell types in which a given peak occurs. Only the 12 assays that have been performed in more than 10 cell types are shown.

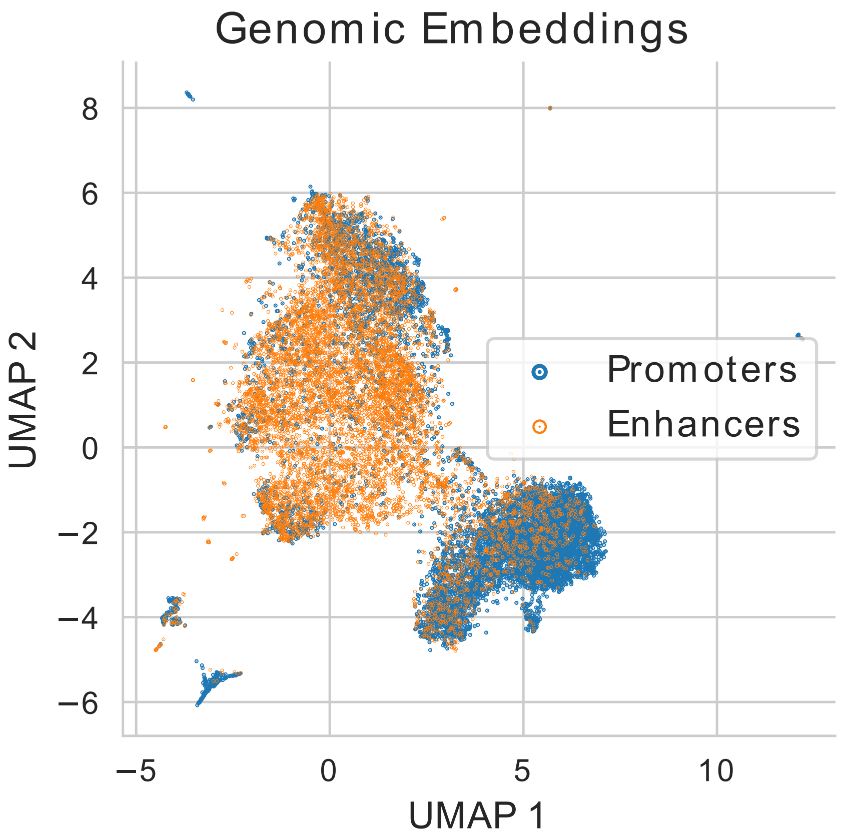

Figure S4: **A projection of Avocado's genome embeddings with a ±2kbp window.** This plot shows the same procedure as Figure 3a, except that the window used here is ±2kbp rather than ±250bp.

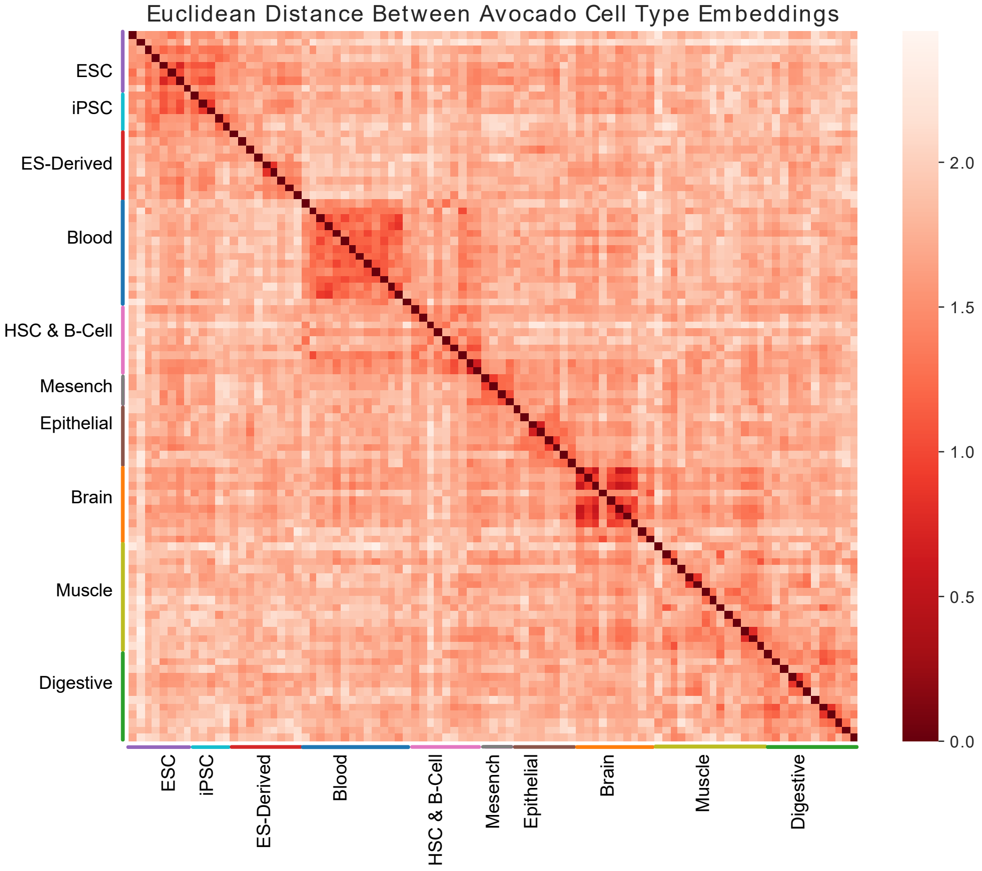

Figure S5: **Euclidean distance matrix between the cell type embeddings learned by Avocado.** The euclidean distances between 93 cell type embeddings learned by Avocado and inspected in Figure 3d. Cell types are grouped by anatomy type, as denoted on the axes, with anatomy type colored the same as Figure 3d.

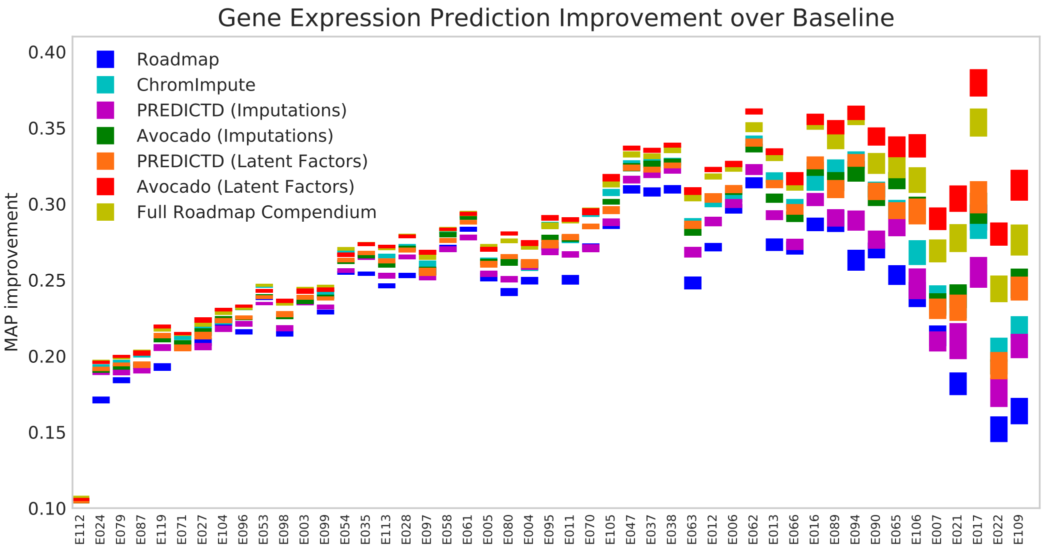

Figure S6: **Relative improvement over a random baseline for each feature set at predicting gene expression.** This plot shows the same values as Figure 5a except that the values for each cell type have the majority baseline subtracted out. This view provides a more detailed look at the relative performance of each of the feature sets, even when the performance of all metrics is high.

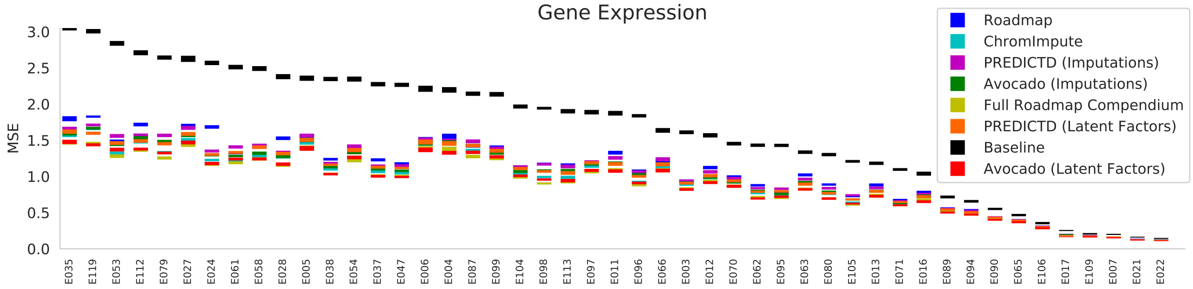

Figure S7: **Performance of machine learning models trained using various feature sets at regressing gene expression values.** This plot shows the performance of models trained in the same manner as those in Figure 5a except that the models are trained on the regression task of predicting gene expression values directly. Accordingly, the models are evaluated using mean squared error rather than average precision.

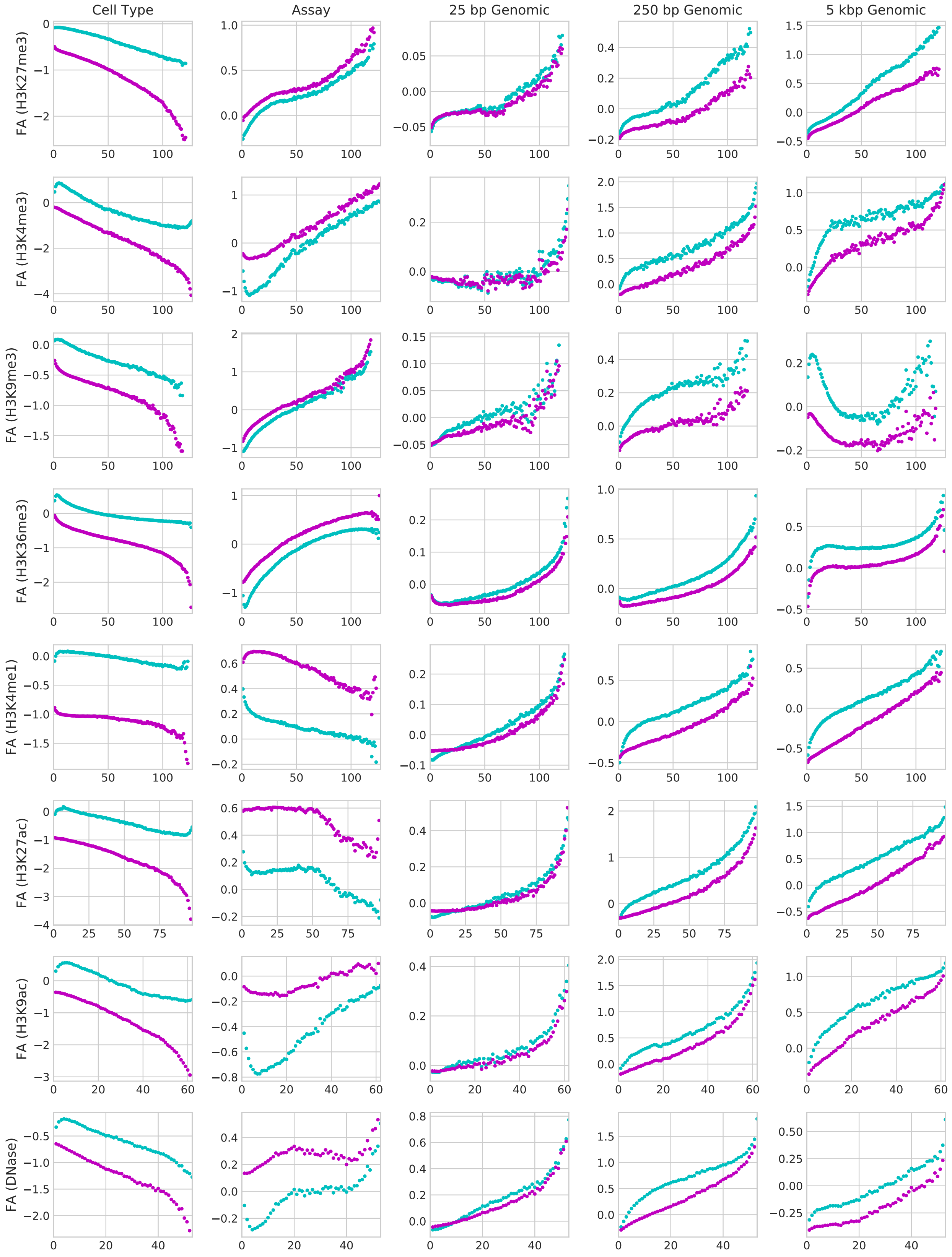

Figure S8: **Feature attribution performed on the Avocado model.** Feature attribution was performed for each position in chromosome 20 across all 1,014 experiments. The results were then aggregated in a manner similar to the analysis of cell-type specific imputations. Instead of calculating the MSE, precision, and recall, instead only the average attribution value is calculated. However, this is done for each of the five model components (the columns). Additionally, the average attribution value is calculated both for those cell types where a peak is exhibited (cyan) and those cell types where a peak is not exhibited (magenta).
