# Supplementary material for: Avocado: a multi-scale deep tensor factorization method learns a latent representation of the human epigenome
Source: Genome Biol. 2020 Mar 30;21:81. doi: 10.1186/s13059-020-01977-6 (PMC7104480; doi:10.1186/s13059-020-01977-6)
Supplement: Supplementary file 4 — Additional file 4 Avocado’s imputed tracks are consistent with known biology. Follow-up analyses showing that the imputations made from Avocado are more globally consistent across pairs and triplets of assays than the imputations made from other models. [file 13059_2020_1977_MOESM4_ESM.pdf]

Additional File 4: Avocado's imputed tracks are consistent with known biology

Jacob Schreiber[1], Timothy Durham[2], Jeffrey Bilmes[1,3], and William Stafford Noble[1,2]

[1]Paul G. Allen School of Computer Science and Engineering, University of Washington, Seattle, USA
[2]Department of Genome Sciences, University of Washington, Seattle, USA
[3]Department of Electrical Engineering, University of Washington, Seattle, USA

November 30, 2019

To better understand the relative behavior of the three imputation methods, we evaluated the imputed measurements for specific histone marks based on their enrichment in functional elements. In particular, H3K4me3 is known to form peaks within transcription start sites (TSSs) and H3K36me3 is known to localize within transcribed genes [1, 2]. We began by extracting the values of H3K4me3 from all TSSs and H3K36me3 from all gene bodies for each cell type. We note that the average H3K4me3 profile across TSSs forms a distinctive bimodal peak (Additional file 4: Figure S1a). Previously, Ernst and Kellis showed that imputed versions of these histone marks exhibit significantly less variation across cell types than the same signal from ChIP-seq tracks, a trend that is also exhibited by PREDICTD and Avocado [3]. An open question is whether this observed reduced variance corresponds to reduction in noise or reduction in true variation among cell types.

To address this question, we first test whether the observed reduction in variation preserves cellular variation by calculating the rank correlation across cell types between imputed signal and ChIP-seq signal according to the area under each cell types' average mark profile (Additional file 4: Fig S1a/b). This analysis shows that Avocado preserves the ordering of cell types the best in both H3K4me3 and H3K36me3, while still reducing the variation of the signal. In contrast, while ChromImpute reduces the variation across cell types the most, there is almost no correlation of this measurement between the ChromImpute-imputed H3K36me3 signal and the ChIP-seq measurements. We next test whether cellular variation is maintained by re-implementing the PromRecov and GeneRecov performance measures proposed by Ernst and Kellis that measure how well these two marks localize within their respective regions. All three imputation strategies show similar localization of H3K36me3 in gene bodies (Additional file 4: Figure S1c), but Avocado shows the highest localization of H3K4me3 in promoter regions in 23 cell types, and a higher localization than ChromImpute in 87 cell types (Additional file 4: Figure S1d).

	**ChromImpute**	**PREDICTD**	**Avocado**
**H3K4me4 - H3K27me3**	0.3566	0.3448	**0.3219**
**H3K4me1 - H3K27me3**	0.3664	0.3150	**0.3059**
**H3K36me3 - RNAseq**	0.2425	0.1531	**0.1464**
**H3K4me3 - H3K36me3**	0.2789	0.2887	**0.2713**
**H3K27me3 - H3K36me3**	0.3163	0.2838	**0.2735**

Table S1: Evaluation of ChromImpute, PREDICTD, and Avocado at reconstructing relationships between different histone marks across the genome according to the mean absolute error. The best result is in boldface for each comparison.

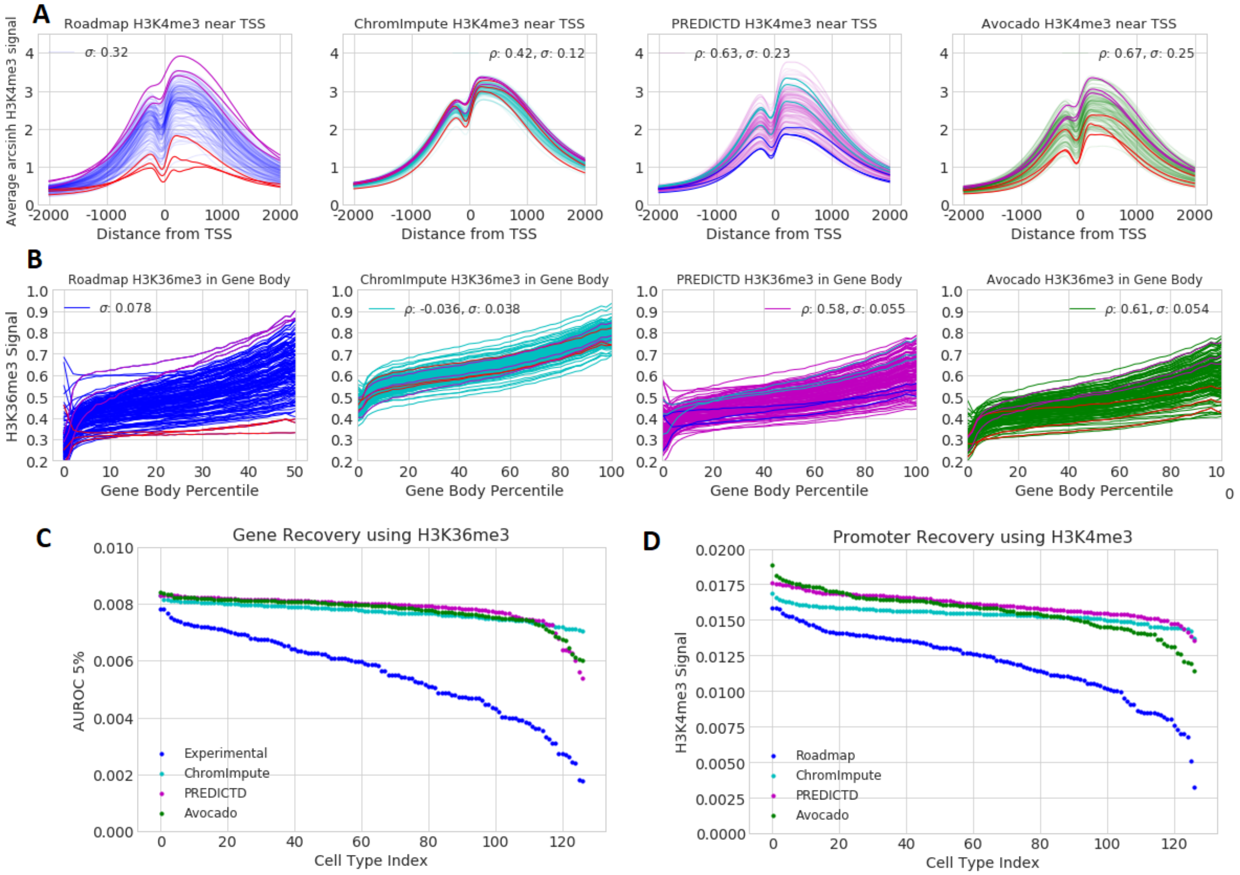

Figure S1: **Aggregate measures of H3K4me3 and H3K36me3 in ChIP-seq experiments and across imputation methods.** (a) H3K4me3 signal in TSSs. Each line displays the average H3K4me3 signal across all TSSs in chromosomes 1-22 for a single cell type after accounting for strand orientation of the gene. The variance of the signal across all cell types at each position is calculated and then averaged (σ). The area under each line is used to define a ranking, and the spearman correlation (ρ) is calculated between each of the three imputation approaches and the ChIP-seq data. To show how the imputation methods alter ordering, the three cell lines with the highest and lowest ChIP-seq signal using this method are colored magenta and red, respectively. In the PREDICTD panels the three with the highest signal are colored cyan and the three with the lowest signal are colored blue. (b) H3K36me3 signal in gene bodies, taking into account the strand orientation of the gene. Measurements are calculated in the same manner as H3K4me3. (c) The GeneRecov performance measure for each cell type. This performance measure quantifies how well H3K36me3 localizes in gene bodies across cell types. It is the area under the ROC curve at 5% FPR when using H3K36me3 to predict gene bodies across chromosomes 1 through 22. (d) The PromRecov performance measure for each cell type. This performance measure quantifies how well H3K4me3 localizes in promoter regions across cell types. It is the area under the ROC curve at 5% FPR when using H3K4me3 to predict promoters across chromosomes 1 through 22.

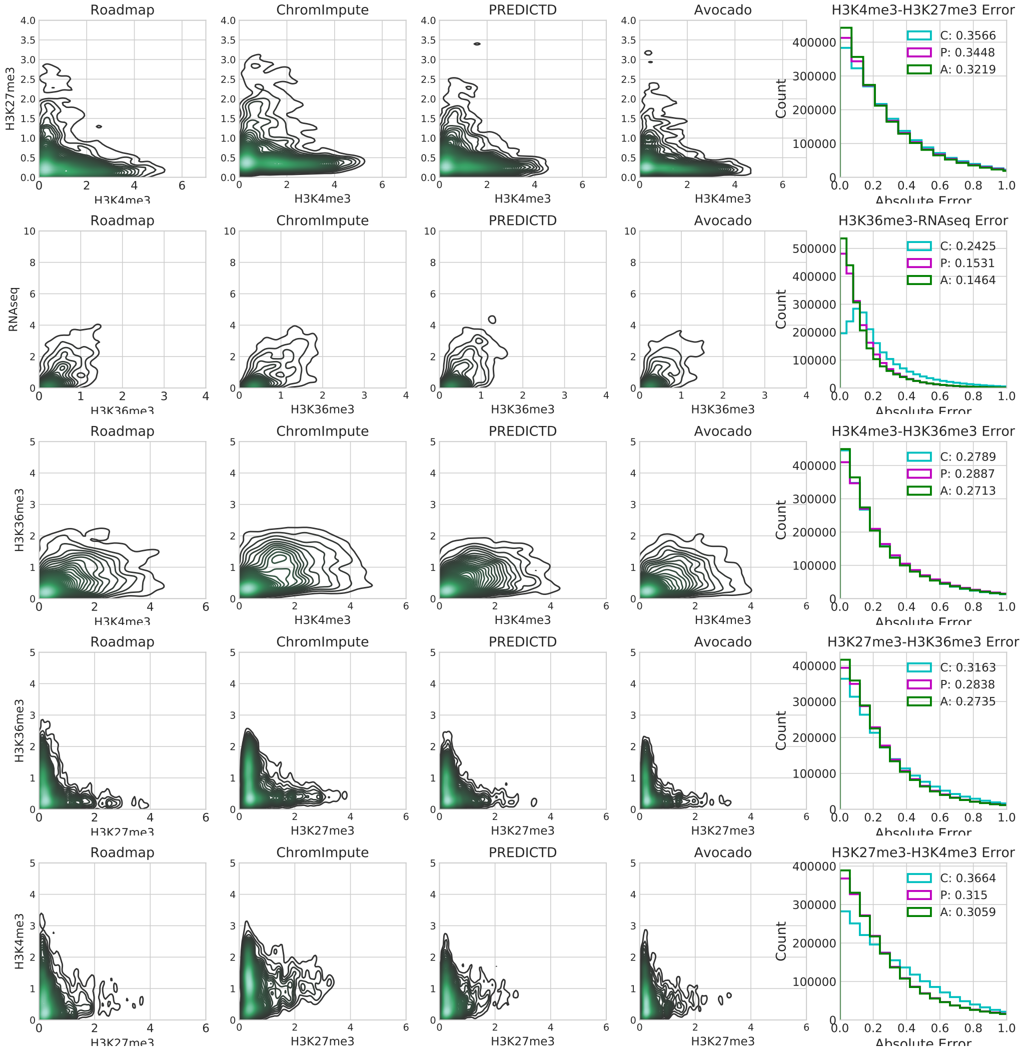

Figure S2: **The relationships between pairs of histone modifications.** These panels show, going from left to right, the signal values in the Roadmap compendium, the imputed signal values from ChromImpute, imputed signal values from PREDICTD, the imputed signal values from Avocado, and the distribution of the absolute error in reconstructing the relationship. In the rightmost panels the legend denotes ChromImpute as C, PREDICTD as P, and Avocado as A. Because each plot contains over 2 million samples, the contour plots are generated on a randomly selected one thousandth of the data, though the error histogram is generated from the full set of samples.

To expand on this investigation, we then looked at each techniques' ability to reconstruct relationships among multiple histone marks at the same locus in the genome. We began by looking at the signal values of repressive mark H3K27me3 and the activating mark H3K4me3 in promoter regions, because the two marks tend not to co-localize in differentiated cells (Additional file 4: Figure S2). To quantitatively evaluate this relationship, we calculate the difference between H3K4me3 and H3K27me3 across all 127 cell lines for all promoter regions and calculate the mean absolute error (MAE) between the ChIP-seq signal and the corresponding imputed tracks. This performance measure measures how well the imputation strategies are able to preserve the difference between the two marks. We find that Avocado achieves a lower MAE at reconstructing this relationship than either other method (Additional file 4: Table S1). We also verified that Avocado does a better job than the other two imputation methods at capturing a lack of correlation between unrelated marks (Additional file 4: Figure S2), such as the repressive mark H3K27me3 and enhancer-associated mark H3K4me1 (Additional file 4: Table S1).

We then consider how well the methods can reconstruct the relationship between H3K36me3, a mark typically associated with active gene transcription, and RNA-seq measurements in gene bodies. We restricted our comparison to 47 cell types in which RNA-seq measurements were available from the Roadmap consortium. In this analysis, Avocado captures the relationship the best, and ChromImpute the worst. (Additional file 4: Figure S2).

We then considered relationships across both histone marks and genomic loci, focusing on the relationship between marks in the promoter and the gene body. Specifically, we consider the relationship between H3K4me3 in the promoter region with H3K36me3 in the gene body, because an enrichment of the activating mark should lead to higher levels of the transcription-associated mark. Likewise, we would expect that an enrichment in H3K27me3 in the promoter region should lead to a depletion of H3K36me3 in the gene body. A priori, we expect that ChromImpute and Avocado would do particularly well at reconstructing these interactions because they both take as input information from many nearby genomic loci, whereas PREDICTD treats each genomic position independently. However, we find that while PREDICTD does the worst at reconstructing the relationship between H3K4me3 and H3K36me3, ChromImpute performs much worse at connecting H3K27me3 and H3k36me3 (Additional file 4: Table S1). Interestingly, despite ChromImpute having an overall negative correlation between H3K27me3 and H3K36me3, as ChromImpute's imputed value of H3K27me3 increases so too does the minimum value of H3K36me3 (Additional file 4: Figure S2). This trend exists to a much lesser extent in the Avocado model, but is not supported by the ChIP-seq signal.

References

[1] Anshul Kundaje, Wouter Meuleman, Jason Ernst, Misha Bilenky, Angela Yen, Alireza Heravi-Moussavi, Pouya Kheradpour, Zhizhuo Zhang, Jianrong Wang, and Michael J Ziller. Integrative analysis of 111 reference human epigenomes. *Nature*, 518(7539):317–330, 2015.

[2] ENCODE Project Consortium. An integrated encyclopedia of DNA elements in the human genome. *Nature*, 489:57–74, 2012.

[3] Jason Ernst and Manolis Kellis. Large-scale imputation of epigenomic datasets for systematic annotation of diverse human tissues. *Nature Biotechnology*, 33(4):364–376, 2015.
