# Supplementary material for: Avocado: a multi-scale deep tensor factorization method learns a latent representation of the human epigenome
Source: Genome Biol. 2020 Mar 30;21:81. doi: 10.1186/s13059-020-01977-6 (PMC7104480; doi:10.1186/s13059-020-01977-6)
Supplement: Supplementary file 5 — Additional file 5 Inspection of genome embeddings. Follow-up analyses inspecting the projection of the genome embeddings from enhancer and promoter regions to investigate the third cluster made up of low-signal regions. [file 13059_2020_1977_MOESM5_ESM.pdf]

# Additional File 5: Inspection of genome embeddings

Jacob Schreiber[1], Timothy Durham[2], Jeffrey Bilmes[1,3], and William Stafford Noble[1,2]

[1]Paul G. Allen School of Computer Science and Engineering, University of Washington, Seattle, USA
[2]Department of Genome Sciences, University of Washington, Seattle, USA
[3]Department of Electrical Engineering, University of Washington, Seattle, USA

November 30, 2019

We inspected the three clusters in Figure 3a to better understand the types of loci that each cluster contains. Specifically, we wanted to understand whether the loci comprising the "mixed" cluster exhibited low average signal across all cell types because the loci were active in a very cell type-specific manner, or simply always demonstrated low signal. We began with the epigenomic signal ±2kbp around each locus in each cell type. This data was then divided into four sets: (1) H3K4me3 in promoters, (2) H3K27ac in promoters, (3) H3K4me3 in enhancers, and (4) H3K27ac in enhancers. We averaged each signal across all cell types but partitioned the loci based on which cluster from Figure 3a they were a part of (Figure 3b). We will refer to the epigenomic signal ±2kbp around a locus in a particular cell type as a "profile", so that it is not confused with the term "locus". Thus, each locus has one profile per cell type.

Next, we applied k-means clustering to each of these four sets separately to split the profiles into "high-signal" profiles and "low-signal" profiles. We adopt this terminology rather than the more traditional term "cluster" so as to not confuse these with the three clusters from Figure 3a. As expected, the average high-signal profile shows patterns commonly seen with active functional elements, whereas the average low-signal profile shows almost no signal (Additional file 5: Figure S1). Furthermore, the average high-signal profile looks consistent across all three clusters, giving initial evidence that the mixed cluster is not made up exclusively of low-signal profiles.

Lastly, we adopted a more comprehensive view of the signal partitions by examining the number of high-signal profiles per locus. For each set, we examined the partition that each locus was assigned to in each cell type (i.e. each profile). We then summed the number of cell types that exhibited high-signal profiles per locus (Additional file 5: Figure S2). We found that, although the mixed cluster appeared to be made up predominately of loci that exhibit low signal in all cell types, there are indeed many loci that exhibit high signal in a very cell type-specific manner. It is likely that, at loci that exhibit lower signal in all cell types, a weaker regulatory signal is sufficient for regulatory function. These observations explain why a model like Avocado, which is trained using the signal strength directly, groups these loci together, separate from either the promoter or the enhancer cluster.

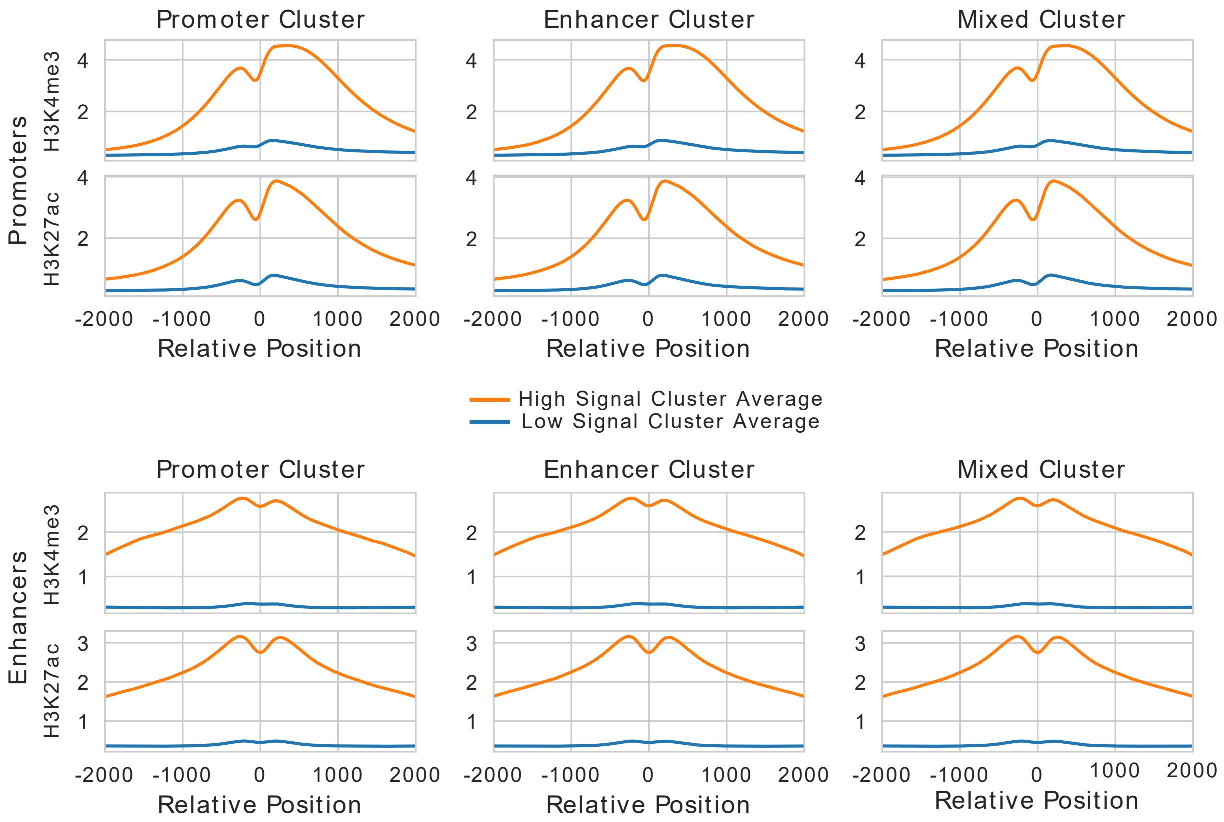


Figure S1: **Average epigenomic profiles of clustered loci.** The average epigenomic activity of loci clustered into a "high" signal cluster (orange) and a "low" signal cluster (blue). The average profile for these clusters is shown for each of the three clusters (columns) and four sets (rows)

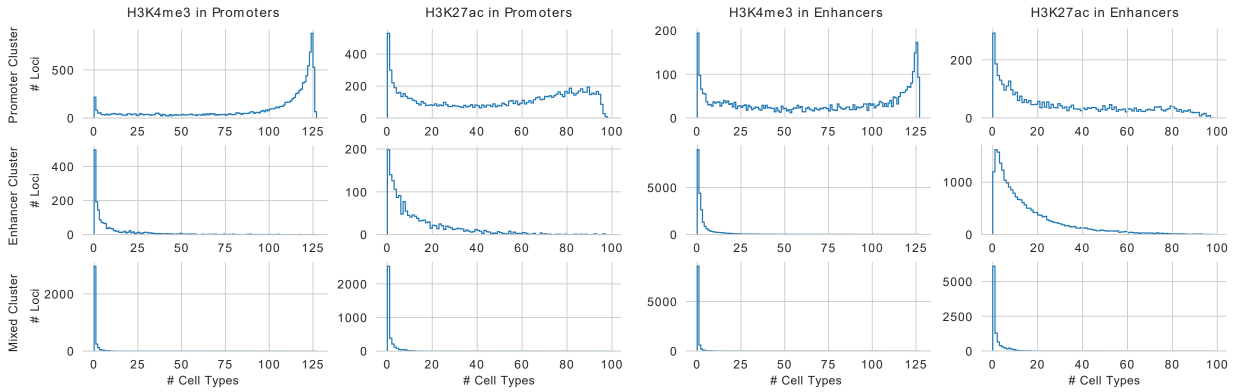


Figure S2: **Cell type specificity of profile signals** Each panel shows a distribution of the number of cell types that each profile exhibits high signal. These profiles come from each of the four sets (columns) and are partitioned according to the three original clusters (rows).
