# Supplementary material for: Avocado: a multi-scale deep tensor factorization method learns a latent representation of the human epigenome
Source: Genome Biol. 2020 Mar 30;21:81. doi: 10.1186/s13059-020-01977-6 (PMC7104480; doi:10.1186/s13059-020-01977-6)
Supplement: Supplementary file 6 — Additional file 6 Promoter-enhancer interaction data set. A description of the manner that the promoter-enhancer interaction data set was constructed from the original TargetFinder data set. [file 13059_2020_1977_MOESM6_ESM.pdf]

Additional File 6: Promoter-enhancer interaction data set

Jacob Schreiber[1], Timothy Durham[2], Jeffrey Bilmes[1,3], and William Stafford Noble[1,2]

[1]Paul G. Allen School of Computer Science and Engineering, University of Washington, Seattle, USA
[2]Department of Genome Sciences, University of Washington, Seattle, USA
[3]Department of Electrical Engineering, University of Washington, Seattle, USA

November 30, 2019

The set of promoter-enhancer interactions used to evaluate the TargetFinder model [1] has been recently shown to contain biases related to the pairwise nature of the task [2]. The bias arises for two reasons. First, the data set includes features derived from the window between the promoter and the enhancer, and these features are highly correlated between examples whose windows overlap. This correlation leads to a leakage of information when regions of the genome are in windows of examples in both the training and the test set. Fortunately, this issue can be easily corrected by simply removing the problematic features. The second issue is that when the data set was constructed, an equal number of positive and negative interactions were sampled at each genomic distance. Consequently, many promoters occur repeatedly and only in the context of a negative interaction. When promoter-enhancer pairs are randomly assigned to both the training and test sets, as is the case with the TargetFinder model, then a sufficiently complicated model can simply memorize these repeated promoters as never interacting. These issues are described more thoroughly by Xi and Beer [2].

To construct a data set without these biases, we choose the simple approach of filtering out interactions such that each promoter occurs only once in each cell type. While we do not also enforce that enhancers can only occur once, we greedily select pairs where the enhancer has not yet been part of an example. This approach yields a data set with 27,048 interactions across all four cell types in chromosomes 1 through 22, where each interaction corresponds to a unique promoter in its cell type. Among these interactions, nearly all (26,707) have unique enhancers as well; 158 enhancers are seen twice, 7 are seen 3 times, and 1 is seen four times. After this filtering step, IMR90 has 4,702 pairs, of which 82 are positive interactions; GM12878 has 7,881 pairs, of which 181 are positive interactions; HeLa-S3 has 7,060 pairs, of which 121 are positive interactions; and K562 has 7,405 pairs, of which 145 are positive interactions. Promoters and enhancers

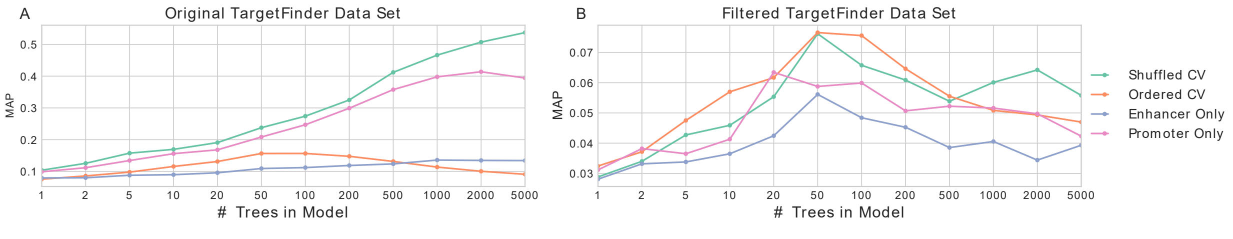

[b]

Figure S1: **Model performance on the original and filtered TargetFinder data sets.** (A) The performance of gradient boosting classifiers on the TargetFinder data set split by randomly assigning interactions to folds (cyan) or ordering interactions by genomic coordinate and then splitting into consecutive blocks (orange). Further, when randomly assigning interactions to folds, the performance is shown when using only features from the enhancer (blue) and when using features only from the promoter (pink). (B) Similar to (A), but on the new filtered data set.

were defined by Whalen *et al.* to be those identified using combined Segway and ChromHMM annotations for the respective cell types. Further, promoters were then filtered to be only those in GENCODEv19 that were actively transcribed (mean FPKM > 0.3 and IDR < 0.1 using corresponding ENCODE RNA-seq data for each cell type). Thus, both the positive and negative sets for our predictive tasks were defined on active regulatory elements.

We verify that the source of bias has been removed using the same techniques used by Xi and Beer [2]. First, we plot the performance of gradient boosting models with an increasing number of trees evaluated using five-fold cross-validation with examples randomly assigned to folds. We observe a steadily increasing performance on the original data set, similar to that reported by Whalen et al. [1], but not on the new data set (Additional file 6: Figure S1, orange). Next, we sort examples based on their genomic coordinates and assigned samples to folds based on this ordering. We observe the same diminished performance on the original data set in comparison to random splitting that was observed by Xi and Beer (blue), but similar performance on the new data set compared to random splitting. Lastly, to confirm that this issue is related to memorizing which promoters never interact, we train models using features from only the promoter (green) or the enhancer (brown). We observe similar performance in the original data set when using all features or only using features derived from the promoter region. However, we do not observe this trend in the new data set. Taken together, these results confirm that the new data set does not exhibit the same issue as the original data set used by Whalen et al [1].

References

[1] S. Whalen, R. M. Truty, and K. S. Pollard. Enhancer-promoter interactions are encoded by complex genomic signatures on looping chromatin. *Nature Genetics*, 48:488–496, 2016.

[2] W. Xi and M.A. Beer. Local epigenomic state cannot discriminate interacting and non-interacting enhancer-promoter pairs with high accuracy. *PLOS Computational Biology*, 14(12):1–7, 2018.
