# Supplementary material for: Avocado: a multi-scale deep tensor factorization method learns a latent representation of the human epigenome
Source: Genome Biol. 2020 Mar 30;21:81. doi: 10.1186/s13059-020-01977-6 (PMC7104480; doi:10.1186/s13059-020-01977-6)
Supplement: Supplementary file 7 — Additional file 7 Subsampling for first stage of training. Follow-up analyis showing that using the ENCODE Pilot Regions for the first step of the Avocado training procedure does not lead to significantly worse imputations than another subsample. [file 13059_2020_1977_MOESM7_ESM.pdf]

Additional File 7

Jacob Schreiber[1], Timothy Durham[2], Jeffrey Bilmes[1,3], and William Stafford Noble[1,2]

[1]Paul G. Allen School of Computer Science and Engineering, University of Washington, Seattle, USA
[2]Department of Genome Sciences, University of Washington, Seattle, USA
[3]Department of Electrical Engineering, University of Washington, Seattle, USA

November 30, 2019

We tested whether the performance of Avocado was sensitive to the set of regions used in the initial training step when the assay embeddings, cell type embeddings, and neural network weights are learned. Our primary model uses the ENCODE Pilot Regions for this step. In this supplementary analysis, we trained five additional models using signal from contiguous blocks of the same size as the ENCODE Pilot Regions extracted from the centers of chromosomes 1 through 5. Then, for each of the five models, we froze the assay embeddings, cell type embeddings, and the neural network weights, and we fit the genome factors for chromosome 16. These models were each trained using experiments from four of the five folds from the five-fold cross-validation in both of these steps and then evaluated based on their ability to impute the remaining fifth fold of experiments in chromosome 16. We found that the model trained using the ENCODE Pilot Regions were similar to those trained using the contiguous blocks of the genome.

**Step 1 Trained On**	**Step 2 Trained On**	**Test Set MSE**
ENCODE Pilot Regions	chr16	**0.0733**
chr1	chr16	**0.0755**
chr2	chr16	**0.0689**
chr3	chr16	**0.0700**
chr4	chr16	**0.0711**
chr5	chr16	**0.0770**

Table S1: Performance of six models when evaluated using the same region—chromosome 16—but trained using different regions for the initial training step.
