# Supplementary material for: Avocado: a multi-scale deep tensor factorization method learns a latent representation of the human epigenome
Source: Genome Biol. 2020 Mar 30;21:81. doi: 10.1186/s13059-020-01977-6 (PMC7104480; doi:10.1186/s13059-020-01977-6)
Supplement: Supplementary file 8 — Additional file 8 The review history. [file 13059_2020_1977_MOESM8_ESM.docx]

Review History

**First round of review**

**Reviewer 1**

**Comments to author:**

In this paper, the authors report a method that impute the missing values of the ENCODE matrix. Specifically, the input data are: cell types, encoded as binary labels; assay types, encoded as binary label, chromosome positions, each encoded as a binary label.

I have two major comments on the methodology:

Firstly, encoding every position of the chromosomes as an input is not an efficient method. As a matter of fact, this algorithm cannot run on the entire genome, as the authors have stated. It is not a scalable method. There are many other approaches to encode the information which can help the algorithm to take advantage of the information from the whole genome.

Secondly, the authors used MSE to call back the model and evaluate the model. This approach seems to work due to an artifact or bias in the ENCODE data generation and design, in which each lab tends to accomplish the experiments of a single cell line across multiple assays. The differences between the data generated by different labs is what is being learned here. Thus, what is being coded by the cell types in the first step and learned and confirmed by MSE is largely this bias.

**Reviewer 2**

**Comments to author:**

The authors present Avocado, a new method for genome-wide prediction of epigenomic data. Like their previous method PREDICTD, Avocado is based on latent factors for assays, cell types, and genomic position. However, in Avocado, these are the input of a neural network with 2048 neurons in each of two hidden layers. The authors benchmark this novel approach on the same data sets also used for assessing ChromImpute and PREDICTD before. Furthermore, they scrutinize the utility of the latent factors learned by Avocado in different setting. Specifically, they consider embeddings in the different dimensions (genomic position, assays, cell types) and using latent factors as features of different prediction tasks.
The method and results are highly convincing, in general. However, I have a few major concerns regarding the benchmarking procedure and description of the methods, as detailed below.

Major

1. For the performance assessment of the prediction of gene expression (section 2.4.1), the authors consider this as a classification task instead of a regression tasks. As the class labels inherently depend on some threshold, which could be argued about, and as an accurate distinction of highly and mid-to-lowly expressed genes would be interesting as well, I would strongly suggest to also perform this evaluation on the regression task.

2. In section 5.1 the regions considered for different task appear to be inconsistent and not in agreement with what is shown in the figures. Specifically, the authors state that promoters where defined as 2 kbp upstream of the TSS (page 16, line 28) and +-250 bp were used for visualization.
a) Why is only sequence upstream of the TSS considered as promoter for the expression prediction task, while regulatory elements may also appear in 5' UTRs (and also first introns).
b) From the description in section 2.3 it seems that for Figure 3a, +-250 bp have around TSS or enhancer center have been used, whereas -+2 kbp are considered in Figure 3b (in contrast to the description in section 5.1). This distinction should be clear from the description in section 5.1.
c) The choice of +-250 bp in Figure 3a (whereas those clusters are visualized for +-2 kbp in Fig. 3b appears to be quite singular. Hence, I wondered how the UMAP embedding would look like for +-2 kbp.

3. From the companion paper, I learned that an imputation for novel assays or cell types is generally possible without re-training the complete model. As this question came to my mind when reading this manuscript, I would strongly recommend to include a statement regarding this possibility (with reference to the companion paper) to this paper, e.g., in the Discussion section.

4. The general description of the training process in section 5.4 was largely clear. However, it is not obvious how this procedure translated to the cross validation setting. Are both steps (training on pilot, chromosome-wise training of genomic factors) performed in each step of the cross validation procedure (assuming that combinations of assay and cell type are not present in both the training and test partition, see below)?


Minor

5. For the five-fold cross validation in the comparison to PREDICTD and ChromImpute, it remained unclear to me how the partitions have been chosen. I would assume that for each fold, one combination of assay and cell type (across all genomic loci) is present either in the training or in the test partition. However, this should be explicitly stated somewhere in the text (either section 2.2 or Methods).

6. In addition to the curves in Supp. Fig. S3, it would be interesting to see the different performance measures (in analogy to Table 1) split by assay type as supplementary tables.

7. For Figure 2b, the authors state that the ChIP-seq signal is thresholded at a value of 1.44. However, for computing precision and recall, the predictions need to be thresholded as well. Please provide the corresponding threshold in the caption of Figure 2.

8. On page 8, line 32, the authors speculate that the low signal in the mixed cluster is due to very cell type-specific signals. I would second that assumption, but it might be possible to check this assumption by a) computing the profiles of Figure 3b per cell type and region, and b) clustering these according to the profiles. Ideally, this might give insights into the fraction of regions with highly cell type-specific signals.

9. The results presented in Figure 3d are generally convincing, but I wondered if the authors could give an explanation why "digestive", "muscle", and "brain" cell types appear so close together in the embedding.

10. On page 9, line 53, the authors refer to "the average RNA-seq signal". However, I failed to find a definition of "the RNA-seq signal" in this section or the methods section. If currently not present, details should be added either in this section or section 5.1.

11. Unless the authors have arguments against it, I would consider Supp. Fig. S4 more informative than Fig. 5s, and would suggest to swap both figures.

12. In Figure 5 and supplementary figures, the authors use (mean) average precision instead of the area under the precision recall curve (auPRC). As I would consider auPRC the more common measure, I would suggest to use auPRC for assessing classification tasks if no computation issues render this impossible.

13. For the set of PEIs in section 2.4.2, please also give a rough description what kind of regions are included in the negative set of the associated classification task.

14. In Figure 6, the colors used for the different attributions are hard to distinguish. I suggest to use a clearer color scale that also allows colorblind individuals to distinguish the different areas.

15. In the Discussion (page 14, line 34), the authors attribute peaks that appear in exactly one cell type to "experimental noise or technical error". However, on the contrary, such peaks might also be due to true biology and might be "discounted" in just the same way.

16. When I installed Avocado via pip, the required package "numba" was not installed and I had to do so manually.

17. Typos, grammar, etc.
- page 1, line 52: "measuring factor binding" -> "measuring transcription factor binding"
- page 2, line 37: "higher transcription" -> "a higher transcription rate"
- page 4, line 48: please add reference to Durham et al. ([5])
- the term "FIREs" should be explained on first occurrence (page 9, line 13?), but currently is explained only later (page 12, line 23).
- page 9, line 28: "For three of these tasks", these tasks should be listed explicitly
- The caption of Supp. Fig. S4 refers to "Figure 4a", which should be "Figure 5a".
- The caption of Supp. Fig. S4 refers to a "random baseline", whereas in section 2.4, the authors refer to a "majority baseline". Please clarify if both are the same.
- page 9, line 56: it is not completely clear what "these 13 cell types" refers to. Maybe, "the 13 remaining cell types" (of the 47 in total) would be more obvious.
- page 13, lines 43 and 45: "deuodenum" -> "duodenum"
- the last part of the sentence starting on page 13, line 53 ("and variability of positions...") seems to have a grammar issue and could be rephrased.

**Authors Response**

**Point-by-point responses to the reviewers’ comments:**

**Reviewer 1**

In this paper, the authors report a method that impute the missing values of the ENCODE matrix. Specifically, the input data are: cell types, encoded as binary labels; assay types, encoded as binary label, chromosome positions, each encoded as a binary label. I have two major comments on the methodology:

Firstly, encoding every position of the chromosomes as an input is not an eﬃcient method. As a matter of fact, this algorithm cannot run on the entire genome, as the authors have stated. It is not a scalable method. There are many other approaches to encode the information which can help the algorithm to take advantage of the information from the whole genome.

*We are uncertain why the reviewer thinks that the method cannot run on the entiregenome, astheconclusion states that the model is trained to impute genome-wide epigenomics experiments. While it is true that genomic embeddings for the whole genome cannot ﬁt in GPU of today’s memory, Section 5.4 paragraph 2 describes how we overcome this limitation with a two-stage procedure to train a model in a genome-wide manner. Indeed, as we describe in the paper, our comparison to previous work is done on genome-wide imputations, and the downstream tasks involve loci across the entire genome.*

Secondly, the authors used MSE to call back the model and evaluate the model. This approach seems to work due to an artifact or bias in the ENCODE data generation and design, in which each lab tends to accomplish the experiments of a single cell line across multiple assays. The diﬀerences between the data generated by diﬀerent labs is what is being learned here. Thus, what is being coded by the cell types in the ﬁrst step and learned and conﬁrmed by MSE is largely this bias.

*We disagree strongly with the assertions made here by the reviewer. First, the data used in this paper were generated by the Roadmap Epigenomics Consortium, not the ENCODE Consortium. Second, a uniﬁed experimental setup and processing pipeline which can be found at http://www.roadmapepigenomics.org/ protocols was used to process all experiments, regardless of the lab they originated from. Third, it is not true in a consortium like Roadmap Epigenomics or ENCODE that “each lab tends to accomplish the experiments of a single cell line across multiple assays.” In fact, it is generally the opposite, with certain labs focusing on production of a particular type of assay, such as DNase-seq, ChIP-seq, or RNA-seq experiments. Fourth, we found no evidence to support the claim that our model is simply learning biases, and it is unclear to us why using MSE versus some other regression metric would aﬀect whether a model is learning these biases.*

**Reviewer 2**

The authors present Avocado, a new method for genome-wide prediction of epigenomic data. Like their previous method PREDICTD, Avocado is based on latent factors for assays, cell types, and genomic position. However, in Avocado, these are the input of a neural network with 2048 neurons in each of two hidden layers. The authors benchmark this novel approach on the same data sets also used for assessing ChromImpute and PREDICTD before. Furthermore, they scrutinize the utility of the latent factors learned by Avocado in diﬀerent setting. Speciﬁcally, they consider embeddings in the diﬀerent dimensions (genomic position, assays, cell types) and using latent factors as features of diﬀerent prediction tasks. The method and results are highly convincing, in general. However, I have a few major concerns regarding the benchmarking procedure and description of the methods, as detailed below.

*We thank the reviewer for taking the time to carefully examine our work and provide helpful feedback. We have responded to each point below.*

Major

1. For the performance assessment of the prediction of gene expression (section 2.4.1), the authors consider this as a classiﬁcation task instead of a regression tasks. As the class labels inherently depend on some threshold, which could be argued about, and as an accurate distinction of highly and mid-to-lowly expressed genes would be interesting as well, I would strongly suggest to also perform this evaluation on the regression task.

*We have included results from the regression task as Supplementary Figure S7 (Figure 1 here). Largely, we see the same trends as in the classiﬁcation task. We put these results in the supplement, rather than the main text, because treating expression prediction as a classiﬁcation task is both common in the literature [1–3] and consistent with the other tasks that we use for evaluation in that section. Further, some approaches ﬁrst use a classiﬁer to distinguish those genes with no expression from the others before then using a regression model to predict the expression value of those expressed genes [4, 5]. The corresponding modiﬁed text is as follows*

*‘To conﬁrm these results, we reformulated the problem to be a regression task by removing the threshold on the RNA-seq values used to generate binary labels. We observed similar trends as the classiﬁcation task, with the Avocado latent factors yielding the best model in 27 of the 47 cell types, the full Roadmap compendium yielding the best model in 19 of the 47 cell types, and ChromImpute yielding the best model in one cell type (Supplementary Figure S7). In each cell type, the Avocado latent factors outperformed using the cell type-speciﬁc epigenomic data alone.’*

*Figure 1: Performance of machine learning models trained using various feature sets at regressing gene expression values. This plot shows the performance of models trained in the same manner as those in Figure 5a except that the models are trained on the regression task of predicting gene expression values directly. Accordingly, the models are evaluated using mean squared error rather than average precision.*

2. In section 5.1 the regions considered for diﬀerent task appear to be inconsistent and not in agreement with what is shown in the ﬁgures. Speciﬁcally, the authors state that promoters where deﬁned as 2 kbp upstream of the TSS (page 16, line 28) and +-250 bp were used for visualization.

a) Why is only sequence upstream of the TSS considered as promoter for the expression prediction task, while regulatory elements may also appear in 5’ UTRs (and also ﬁrst introns).

*We agree with the reviewer that regulatory elements can occur after the TSS, and that one might want to use a much larger window (potentially explicitly including gene annotations such as introns) if the goal were to build a state-of-the-art model of gene expression. However, as stated in the manuscript, our goal here is to evaluate the relative utility of various representations of genomic loci. We originally selected the upstream window to highlight epigenomic activity in a region that was involved in regulating gene expression but generally was not itself transcribed. The histone modiﬁcations that we consider are usually enriched on both sides of the TSS when present [6] and in C. elegans have been found to be similarly predictive of gene expression before the TSS and after it (Figure 4C in Cheng et al. [1]). Unfortunately, there is no standard window size to use for promoters when building predictive models of gene expression, and previous models have used ±2 kbp [4, 6], ±5 kbp [3, 7], ±4 kbp [1], 8 kbp upstream to 2 kbp downstream [8], and ±10 kbp [9].*

b) From the description in section 2.3 it seems that for Figure 3a, +-250 bp have around TSS or enhancer center have been used, whereas -+2 kbp are considered in Figure 3b (in contrast to the description in section 5.1). This distinction should be clear from the description in section 5.1.

*In both cases, we use ±250 bp to deﬁne enhancers and promoters, as described in Section 5.1. The only diﬀerence is that, in Figure 3b, we show the enhancer and promoter along with the region surrounding it. We have amended the caption to Figure 3b to state that the proﬁles are “extended out ±2kb to show additional context.”*

c) The choice of +-250 bp in Figure 3a (whereas those clusters are visualized for +-2 kbp in Fig. 3b appears to be quite singular. Hence, I wondered how the UMAP embedding would look like for +-2 kbp.

*We have generated a UMAP embedding for windows of size ±2 kbp and included them as Supplementary Figure S4 (Figure 2 here). The overall trends are quite similar. We have also added the following text.*

*This ±2 kbp window is larger than the ±250 bp window used for the genomic embedding projection in order to give additional epigenomic context, but we found that projecting genomic embeddings using a ±2 kbp window gave similar results (Supplementary Figure S4).*

3.From the companion paper, I learned that an imputation for novel assays or cell types is generally possible without re-training the complete model. As this question came to my mind when reading this manuscript, I would strongly recommend to include a statement regarding this possibility (with reference to the companion paper) to this paper, e.g., in the Discussion section.

*We agree with the reviewer, and we have added a paragraph to the discussion referencing our companion paper and indicating that this is a possibility.*

*‘Finally, we anticipate that researchers may wish to extend the imputation abilities of Avocado to a new cell type or assay using their own experimental data but lack the computational resources to retrain Avocado from scratch. In follow-up work, we describe a simple transfer learning approach for adding in new cell types or assays to a pretrained Avocado model [10]. This approach involves freezing the parameters of a pretrained model and ﬁtting only the new cell type or assay factors. Our analysis suggests that one can achieve good quality imputations with as little as a single track of training data in a given biosample. Further, because very few parameters need to be trained, this process can be done without relying on a GPU.’*

*Figure 2: A projection of Avocado’s genome embeddings with a ±2kbp window. This plot shows the same procedure as Figure 3a, except that the window used here is ±2kbp rather than ±250bp.*

4. The general description of the training process in section 5.4 was largely clear. However, it is not obvious how this procedure translated to the cross validation setting. Are both steps (training on pilot, chromosome-wise training of genomic factors) performed in each step of the cross validation procedure (assuming that combinations of assay and cell type are not present in both the training and test partition, see below)

*We have amended the text to clarify how training is performed in the cross-validation setting.*

*‘Both of the stages involve the same set of training experiments. During cross-validation this procedure is repeated separately for each fold.’*

Minor

5. For the ﬁve-fold cross validation in the comparison to PREDICTD and ChromImpute, it remained unclear to me how the partitions have been chosen. I would assume that for each fold, one combination of assay and cell type (across all genomic loci) is present either in the training or in the test partition. However, this should be explicitly stated somewhere in the text (either section 2.2 or Methods).

*The reviewer is correct in their assumption. We have amended the description in Section 2.2 to clarify how the cross-validation was performed.*

*‘Imputations from Avocado and PREDICTD were made using a ﬁve-fold cross validation approach where the folds used for Avocado were the same as those used for PREDICTD. ChromImpute used leave-one-out validation. In each case, signal values across the entire genome were used either for training or testing.’*

6. In addition to the curves in Supp. Fig. S3, it would be interesting to see the diﬀerent performance measures (in analogy to Table 1) split by assay type as supplementary tables.

*We have added these tables to the supplement as Additional File 1.*

7. For Figure 2b, the authors state that the ChIP-seq signal is thresholded at a value of 1.44. However, for computing precision and recall, the predictions need to be thresholded as well. Please provide the corresponding threshold in the caption of Figure 2.

*We apologize for the confusion. The imputations were thresholded at a value of 1.44. The true labels that were used were MACS2 peak calls, which are similar to simply thresholding the signal, but more robust to noise. We have clariﬁed the caption.*

*‘Precision and recall are computed by thresholding the imputations at 1.44 and comparing to MACS2 narrow peak calls on the corresponding experimental signal. In the plots, the series labeled “Roadmap” use the experimental Roadmap data, likewise thresholded at 1.44, and compared to MACS2 narrow peak calls.’*

9. The results presented in Figure 3d are generally convincing, but I wondered if the authors could give an explanation why “digestive", “muscle", and “brain" cell types appear so close together in the embedding.

*The most pronounced trend in the cell type embedding is the distinction between stem cells and diﬀerentiated cells. This distinction likely arises because the epigenomic diﬀerences among these types of cells dominate the projection. To investigate the reviewer’s question, we inspected the distances used to calculate the UMAP projection (Figure 3 in this document). We observed a block diagonal structure corresponding to each of the anatomy types, but also a weaker block structure separating brain, muscle, and digestive cells from the others.*

*‘These results are supported by a direct inspection of the Euclidean distances used to make the plot, which show similar clusterings by anatomy type (Supplemental Figure S5).’*

*Figure 3: Euclidean distance matrix between the cell type embeddings learned by Avocado. The Euclidean distances between 93 cell type embeddings learned by Avocado and inspected in Figure 3 in the paper. Cell types are grouped by anatomy type, as denoted on the axes, with anatomy type colored the same as Figure 3.*

10. On page 9, line 53, the authors refer to “the average RNA-seq signal.” However, I failed to ﬁnd a deﬁnition of “the RNA-seq signal" in this section or the methods section. If currently not present, details should be added either in this section or section 5.1.

*We have amended the description to now read as the following.*

*‘Genes are considered to be active in a cell if the average normalized read-count value from an RNA-seq experiment across the gene body is greater than 0.5 (see Methods).’*

*We have also expanded the description in the Methods section to read as follows:*

*‘RNA-seq bigwigs containing unstranded normalized read counts across the entire genome for 47 cell types were also downloaded for the purpose of downstream analyses, rather than for inclusion in the imputation task.’*

11. Unless the authors have arguments against it, I would consider Supp. Fig. S4 more informative than Fig. 5s, and would suggest to swap both ﬁgures.

*Our reasoning for keeping Figure 5 is that it is easier to interpret all of the plots in Figure 5 together when all plots have the same y-axis and compare the same feature sets. Supplementary Figure S6 (previously Supplementary Figure S4) contains only a subset of the feature sets and has a diﬀerent y-axis.*

12. In Figure 5 and supplementary ﬁgures, the authors use (mean) average precision instead of the area under the precision recall curve (auPRC). As I would consider auPRC the more common measure, I would suggest to use auPRC for assessing classiﬁcation tasks if no computation issues render this impossible.

*The auPRC can be calculated in a number of ways, and doing so accurately is non-trivial because a linear interpolation between adjacent points is overly optimistic [11]. The average precision is simply a way to calculate auPRC that is not overly optimistic. This is why, for example, it is the preferred implementation in scikit-learn. We indicate this in the text, copied below.*

*‘This evaluation is carried out in a 20-fold cross validation setting in each cell type individually, and we report the mean average precision (MAP), which is one technique for calculating the area under a precision-recall curve, across all 20 folds.’*

13. For the set of PEIs in section 2.4.2, please also give a rough description what kind of regions are included in the negative set of the associated classiﬁcation task.

*We have included a more detailed description of the regions used in our PEI task in the Supplementary Note:*

*‘Promoters and enhancers were deﬁned by Whalen et al. to be those identiﬁed using combined Segway and ChromHMM annotations for the respective cell types. Further, promoters were then ﬁltered to be only those in GENCODEv19 that were actively transcribed (mean FPKM > 0.3 and IDR < 0.1 using corresponding ENCODE RNA-seq data for each cell type). Thus, both the positive and negative sets for our predictive tasks were deﬁned on active regulatory elements.’*

14. In Figure 6, the colors used for the diﬀerent attributions are hard to distinguish. I suggest to use a clearer color scale that also allows colorblind individuals to distinguish the diﬀerent areas.

*We have changed the red colored 250bp attributions to be magenta colored, and the magenta colored 25 bp attributions to be dark blue. Switching red for magenta should make the plots more easily distinguishable. See Figure 4 here*

*Figure 4: The predicted H3K4me3 signal and corresponding attributions for two cell types in the same region of chromosome 20. (a) The prediction and attributions for GM12878, where a tall peak on the right is paired with two much smaller peaks to the left. Many short regions have a positive genomic attribution but a negative cell type attribution that masks them. (b) The prediction and attributions for duodenum smooth muscle. A prominent peak is now predicted on the left, corresponding with a swap from a negative cell type attribution to a positive one. The same short regions that previously were masked by the cell type attributions now have positive cell type attributions and exhibit peaks in the imputed signal.*

15. In the Discussion (page 14, line 34), the authors attribute peaks that appear in exactly one cell type to “experimental noise or technical error". However, on the contrary, such peaks might also be due to true biology and might be “discounted" in just the same way.

*We agree with this point, and we have modiﬁed the discussion accordingly:*

*‘On the other hand, when the discounted peaks correspond to real biology that is simply very cell type-speciﬁc, this tendency may be a source of error.’*

16. When I installed Avocado via pip, the required package “numba" was not installed and I had to do so manually.

*We have removed the reference to numba from the code, as numba was not actually being used.*

17. Typos, grammar, etc.

- page 1, line 52: “measuring factor binding" → “measuring transcription factor binding"

- page 2, line 37: “higher transcription" → “a higher transcription rate"

- page 4, line 48: please add reference to Durham et al. ([5])

- the term “FIREs" should be explained on ﬁrst occurrence (page 9, line 13?), but currently is explained only later (page 12, line 23).

- page 9, line 28: “For three of these tasks", these tasks should be listed explicitly - The caption of Supp. Fig. S4 refers to “Figure 4a", which should be “Figure 5a".

- The caption of Supp. Fig. S4 refers to a “random baseline", whereas in section 2.4, the authors refer to a “majority baseline". Please clarify if both are the same.

- page 9, line 56: it is not completely clear what “these 13 cell types" refers to. Maybe, “the 13 remaining cell types" (of the 47 in total) would be more obvious.

- page 13, lines 43 and 45: “deuodenum" → “duodenum"

- the last part of the sentence starting on page 13, line 53 (“and variability of positions...") seems to have a grammar issue and could be rephrased.

*We thank the reviewer for catching these issues. We have corrected them in the text.*

***References***

*[1] C. Cheng, K. K. Yan, K. Y. Yip, J. Rozowsky, R. Alexander, C. Shou, and M. Gerstein. A statistical framework for modeling gene expression using chromatin features and application to modENCODE datasets. Genome Biology, 12(2):R15, 2011.*

*[2] R. Singh, J. Lanchantin, G. Robins, and Y. Qi. Deepchrome: deep-learning for predicting gene expression from histone modiﬁcations. Bioinformatics, 32(17):i639–i649, 2016.*

*[3] R. Singh, J. Lanchantin, A. Sekhon, and Y. Qi. Attend and predict: Understanding gene regulation by selective attention on chromatin. Advances in Neural Information Processing Systems, pages 6788–6798, 2017.*

*[4] X. Dong, M.C. Greven, A. Kundaje, S. Djebali, J.B. Brown, C. Cheng, T.R. Gingeras, M. Gerstein, R. Guigó, E. Birney, and Z. Weng. Modeling gene expression using chromatin features in various cellular contexts. Genome Biology, 13, Jun 2012.*

*[5] ENCODE Project Consortium. An integrated encyclopedia of DNA elements in the human genome. Nature, 489:57–74, 2012.*

*[6] R. Karlic, H. R.Chung, J. Lasserre, K. Vlahovicek, and M. Vingron. Histone modiﬁcation levels are predictive for gene expression. Proceedings of the National Academy of Sciences of the United States of America, 107(7):2926–2931, 2010.*

*[7] B. Chen, N. S. Kenari, and M. W. Libbrecht. Continuous chromatin state feature annotation of the human epigenome. bioRxiv, page 473017.*

*[8] I. G. Costa, H. G. Roider, T. G. do Rego, and F. de AT de Carvalho. Predicting gene expression in T cell diﬀerentiation from histone modiﬁcations and transcription factor binding aﬃnities by linear mixture models. BMC Bioinformatics, 12, 2011.*

*[9] V. Agarwal and J. Shendure. Predicting mRNA abundance directly from genomic sequence using deep convolutional neural networks. bioRxiv, page 416685, 2018.*

*[10] J. M. Schreiber, J. Bilmes, and W. S. Noble. Completing the ENCODE3 compendium yields accurate imputations across a variety of assays and human biosamples. bioRxiv, 2019.* [*https://www.biorxiv.org/content/10.1101/533273v1*](https://www.biorxiv.org/content/10.1101/533273v1)*.*

*[11] K. Boyd, K. H. Eng, and D. C. Page. Area under the precision-recall curve: Point estimates and conﬁdence intervals. In Machine Learning and Knowledge Discovery in Databases, pages 451–466, Berlin, Heidelberg, 2013. Springer Berlin Heidelberg.*

**Second round of review**

**Reviewer 1**

I found my previous reviews were largely dismissed by the authors. Thus I restate my evaluation.

A major issue of Avocado resides in the overall design of this model. Although the idea of “building one model” seems elegant at first look, it indeed can not work at all in practice. This is because Avocado relies on the embedding of assay names and cell type names to distinguish the genome-wide epigenetic signal. Obversiously, one assay or cell type name only contains e.g. 20 characters, whereas the human genome contains more than 3 billion locations. The authors tried to distinguish a much larger target space (>3 billion) based on a very limited feature space (e.g. 20), which is in fact ridiculous. For example, the chromatin accessibility from DNase-seq is substantially different from a histone modification mark. Yet Avocado is designed to distinguish between these two different genome-wide signals simply based on two names e.g. “DNase-seq” and “H3K9me1” using one neural network. A more reasonable design would be a mark-specific model as proposed in the ChromImpute method.

The predictions made by Avocado, in this form can be meaningless. For example, for a data with sharp peaks, the vast majority of regions have very low values, which results in an average value of 0.01. Using the MSE, as what the author used, a prediction with all scores of 0.01 will have a very good MSE. However, the correlation is zero. This prediction is meaningless.

The signal tracks in Roadmap, either due to processing or other reasons, the mean values and distributions are severely biased by labs and cell lines. Additionally, cell lines especially for histones, are very often assigned by labs. This is not a biological issue, but a technical issue. Using MSE is meaningless for this dataset. And model developed for MSE does not deliver to new, independent cohorts.

**Reviewer 2**

I am happy to state that the authors carefully addressed all of my concerns, leaving only one minor comment I would like to make.

Minor comment:

1. Regarding my previous comment 12 (auPRC), I would like to encourage the authors again to compute auPRC using the interpolation across confusion matrices. There are at least two R packages that are doing the interpolation in the correct manner (precrec and PRROC).

**Authors Response**

**Point-by-point responses to the reviewers’ comments:**

**Reviewer 1**

I found my previous reviews were largely dismissed by the authors. Thus I restate my evaluation.
A major issue of Avocado resides in the overall design of this model. Although the idea of “building one model” seems elegant at first look, it indeed can not work at all in practice. This is because Avocado relies on the embedding of assay names and cell type names to distinguish the genome-wide epigenetic signal. Obversiously, one assay or cell type name only contains e.g. 20 characters, whereas the human genome contains more than 3 billion locations. The authors tried to distinguish a much larger target space (>3 billion) based on a very limited feature space (e.g. 20), which is in fact ridiculous. For example, the chromatin accessibility from DNase-seq is substantially different from a histone modification mark. Yet Avocado is designed to distinguish between these two different genome-wide signals simply based on two names e.g. “DNase-seq” and “H3K9me1” using one neural network. A more reasonable design would be a mark-specific model as proposed in the ChromImpute method.

*The reviewer is mistaken. As stated in the manuscript, Avocado uses the epigenomic data itself rather than the sequence of characters in the cell type or assay type names in order to learn embeddings of assays and cell types. We agree with the reviewer that using the sequence of characters in the assay or cell type names would likely have been a suboptimal approach.*

The predictions made by Avocado, in this form can be meaningless. For example, for a data with sharp peaks, the vast majority of regions have very low values, which results in an average value of 0.01. Using the MSE, as what the author used, a prediction with all scores of 0.01 will have a very good MSE. However, the correlation is zero. This prediction is meaningless.

*We agree with the reviewer that the majority of the genome will have very low signal values. However, our evaluation involves comparing Avocado to baseline models that have been shown to yield useful imputations. We have also partitioned our evaluation based on the signal strength, showing that Avocado makes the best imputations in regions that have consistently high, consistently low, and variable signal across cell types.*

The signal tracks in Roadmap, either due to processing or other reasons, the mean values and distributions are severely biased by labs and cell lines. Additionally, cell lines especially for histones, are very often assigned by labs. This is not a biological issue, but a technical issue. Using MSE is meaningless for this dataset. And model developed for MSE does not deliver to new, independent cohorts.

*We agree that the data contains biases reflective of the generating lab and cell type. However, we believe that our cross-validation procedure shows that the model generalizes between labs. The method is not designed to generalize to new, independent cohorts; rather, it is a method designed to impute missing data within a given, incomplete data tensor. It is not clear to us how the use of MSE as an objective relates to this point.*

**Reviewer 2**

I am happy to state that the authors carefully addressed all of my concerns, leaving only one minor comment I would like to make.

*We thank the reviewer for their helpful comments.*
Minor comment:
1. Regarding my previous comment 12 (auPRC), I would like to encourage the authors again to compute auPRC using the interpolation across confusion matrices. There are at least two R packages that are doing the interpolation in the correct manner (precrec and PRROC).

*We agree with the reviewer that for a small number of points there will likely be an important difference between the average precision and the intepolation method proposed by Davis and Goadrich. However, both approaches are valid ways for calculating the area under the precision-recall curve. Further, the difference in value between the two approaches becomes smaller the more points are used. For example, the PRROC documentation (https://cran.r-project.org/web/packages/PRROC/vignettes/PRROC.pdf) includes an example where even 800 points gives similar results between approaches (0.8777665 and 0.8777661). Given that our examples use thousands of points, we do not anticipate a noticable difference.*
